# Regulation of Steroidal Alkaloid Biosynthesis in Bulbs of *Fritillaria thunbergii* Miq. By Shading and Potassium Application: Integrating Transcriptomics and Metabolomics Analyses

**DOI:** 10.3390/biology14060633

**Published:** 2025-05-29

**Authors:** Jia Liu, Zixuan Zhu, Leran Wang, Qiang Yuan, Honghai Zhu, Xiaoxiao Sheng, Kejie Zhang, Bingbing Liang, Huizhen Jin, Shumin Wang, Wenjun Weng, Hui Wang, Ning Sui

**Affiliations:** 1 School of Pharmaceutical Sciences, Zhejiang Chinese Medical University, Hangzhou 310053, China; 2018201063@njau.edu.cn (J.L.); zzx19861403474@163.com (Z.Z.); yuanqiang0825@sina.com (Q.Y.); 2019201015@njau.edu.cn (H.Z.); shengxiaoxiao2023@163.com (X.S.); 15167538515@163.com (K.Z.); liangbingbing0510@163.com (B.L.); jhz99804@163.com (H.J.); wsm000105@163.com (S.W.); wenjunwork02@163.com (W.W.); 2 Jinhua Academy, Zhejiang Chinese Medical University, Jinhua 321000, China; 3 School of Life Sciences, Zhejiang Chinese Medical University, Hangzhou 310053, China; wanglrnjau@163.com; 4 Qiandao Lake Research Institute, School of Pharmaceutical Sciences, Zhejiang Chinese Medical University, Hangzhou 310053, China

**Keywords:** *Fritillaria thunbergii* Miq., shading, potassium application, steroidal alkaloid, transcription factor

## Abstract

*Fritillaria thunbergii* Miq., a medicinal plant rich in steroidal alkaloids, produces bulbs that clear heat, resolve phlegm, and detoxify. However, excessive yield-oriented cultivation has reduced the number of *F. thunbergii* plants that meet commercial standards. This study explored the effects of potassium application and shading on the bulb biomass and medicinal substance content of *F. thunbergii*. Shading increased the active ingredient content in bulbs by approximately 20.71% but reduced biomass by approximately 17.24%. Fertilization with different potassium concentrations under shading (K1S–K3S) alleviated shading-induced biomass reduction and increased active ingredient accumulation, with the K2S and K3S groups yielding significantly better results than the K1S group. Pharmacological experiments showed that the K2S group exerted the best antitussive, expectorant, and anti-inflammatory effects. Metabolome analysis showed that compared with those in the controls, peiminine, peimine, imperialine, solasodine, and cyclopamine were the most abundant steroidal alkaloids under K2S treatment. Transcriptome analysis identified key genes and biosynthetic pathways for major steroidal alkaloids, namely, farnesyl pyrophosphate synthase (*FtFPS*) involved in steroidal alkaloid biosynthesis. Transcription factor analysis revealed that nine transcription factors predominantly expressed under the K2S treatment might regulate steroidal alkaloid biosynthesis. Furthermore, FtFPS was identified as a hub gene in the co-expression network and was verified to catalyze the biosynthesis of farnesyl pyrophosphate. The interaction between FtFPS and FtAP2/ERF was verified through yeast two-hybrid experiments. These findings offer new insights into the steroidal alkaloid biosynthesis mechanism triggered in *F. thunbergii* by potassium application and shading, supporting ecological strategies to enhance steroidal alkaloid levels in this species.

## Simple Summary

*Fritillaria thunbergii* Miq., a medicinal plant rich in steroidal alkaloids, produces bulbs that clear heat, resolve phlegm, and detoxify. However, excessive yield-oriented cultivation has reduced the number of *F. thunbergii* plants that meet commercial standards. This study explored fertilization with different potassium concentrations under shading. K2S treatment alleviated shading-induced biomass reduction, increased active ingredient accumulation, and pharmacological properties. Metabolome analysis showed peiminine, peimine, imperialine, solasodine, and cyclopamine were the most abundant steroidal alkaloids under K2S treatment. Transcriptome analysis identified key genes and biosynthetic pathways for major steroidal alkaloids. FtFPS was identified as a hub gene in the co-expression network and was verified to catalyze the biosynthesis of farnesyl pyrophosphate. The interaction between FtFPS and FtAP2/ERF was verified through yeast two-hybrid experiments. These findings offer new insights into the steroidal alkaloid biosynthesis mechanism triggered in *F. thunbergii* by potassium application and shading, supporting ecological strategies to enhance steroidal alkaloid levels in this species.

## 1. Introduction

The *Liliaceae* family has been extensively cultivated globally since ancient times [1]. Take *Fritillaria thunbergii* as an example. This herb, belonging to the *Liliaceae* family, is not only utilized in traditional medicine but also finds application in contemporary society. It is frequently processed into various food products such as cakes and porridge, thereby being integrated into daily diets and playing a significant role in preventive health care. Folk medicine, as a vital component of traditional medicine, exhibits significant regional characteristics and is heavily reliant on local natural resources. In contemporary medical practice, folk medicine not only serves as an adjunctive therapy but also demonstrates considerable value in disease prevention [2]. The *Fritillaria* genus is extensively utilized in traditional medicine across various countries, including Japan, India, and China [3,4]. Species of the *Fritillaria* genus are predominantly found in deciduous forests and mountainous regions of Japan, including the Central Mountains of Kyushu and the lowlands of central Honshu [5]. These species play a vital role in Japan’s ecological systems. For example, *Fritillaria borealis*, characterized by its high productivity, serves as a key secondary producer within marine ecosystems [6]. Additionally, *Fritillaria cirrhosa* D. Don, which is primarily distributed in the northwestern region of India, holds significant importance in traditional Indian medicine for its applications in treating cardiovascular and respiratory disorders [7]. In China, the primary cultivation regions of *F. thunbergii* are predominantly located in the coastal provinces of southeastern and central–eastern China, notably Ningbo in Zhejiang Province and Yancheng in Jiangsu Province. These areas are characterized by soil with a relatively high salinity level. *F. thunbergii* bulbs are rich in bioactive compounds, including steroidal alkaloids, polysaccharides, and flavonoids, which exhibit antitussive, expectorant, and anti-inflammatory properties [8]. Rising respiratory diseases driven by climate change have escalated market demand for *F. thunbergii* [9], yet the industry faces bottlenecks due to inefficient cultivation techniques that hinder large-scale production.

Given the depletion of wild *F. thunbergii* resources and ecological conservation policies, commercially available specimens predominantly rely on artificial cultivation systems, such as those using shading treatment, which mimics the light and microclimatic conditions of the native shaded mountainous habitats [10]. Recent studies have demonstrated that moderate shading can enhance the concentration of bioactive components in medicinal plants. The saikosaponin content and total saikosaponin yield in *Bupleurum* reach peak levels under low-light conditions [11]. Similarly, reduced light intensity significantly increases glycyrrhizin and liquiritin accumulation in *Glycyrrhiza* roots [12]. However, excessive shading suppresses photosynthesis, leading to yield losses in crops such as *Paris polyphylla* [13,14] and soybeans in maize–soybean intercropping systems [14,15]. This dual effect—enhanced quality vs. reduced yield—is also evident in *F. thunbergii*. Light intensity is a critical factor influencing photosynthesis in plants. Studies have demonstrated that within the range from the light compensation point to the light saturation point, the net photosynthetic rate of plants increases as light intensity rises. Under low-light conditions, plants improve their light-capturing capacity by increasing chlorophyll concentration or adjusting leaf angles [16]. Furthermore, scientific investigations have demonstrated that light quality significantly influences the accumulation of carotenoids and chlorophyll in plants. The synergistic effect of combined light qualities on carotenoid biosynthesis has also been observed. Specifically, research indicates that the blue and white LED combination enhances total carotenoid levels in *Brassica rapa* (*pak choi*) by 15% compared to red light alone [17]. Additionally, studies conducted on *Arabidopsis* and other crops reveal that blue light indirectly promotes chlorophyll accumulation through the modulation of genes associated with photosynthetic and hormonal pathways [18]. In our previous study, four shading intensities (0%, 50%, 75%, and 95%) were evaluated; the results showed that moderate shading enhances the steroidal alkaloid content in *F. thunbergii* but significantly reduces the bulb biomass and yield [15]. These findings underscore the critical need to optimize shading intensity to reconcile phytochemical quality and agricultural productivity in medicinal plant cultivation.

Farmers in the primary *F. thunbergii* production regions in China frequently overlook potassium fertilization during planting, focusing more on nitrogen fertilization, which leads to both an excessive and inadequate application of potassium fertilizer. Potassium, referred to as the “quality element” [19], has been shown to greatly enhance plant stress resistance and accelerate the growth of underground rhizomes. As a high-potassium-demand crop, *F. thunbergii* requires substantially higher potassium levels for bulb development than cereal crops [20]. Potassium is the most abundant mineral essential for the growth and maturation of medicinal bulb tissues in *F. thunbergii* [21]. Studies demonstrate that potassium fertilization enhances not only nitrogen, potassium, and protein content in onion bulbs but also bioactive constituents such as flavonoids and anthocyanins [22]. Similarly, foliar application of potassium silicate mitigates salt stress in chicory by increasing the carotenoid, total phenolic, and flavonoid contents [23]. Meanwhile, microelements play a crucial role in the accumulation of secondary metabolites in plants. Studies have demonstrated that microelements, such as calcium and magnesium, can exert synergistic effects through mechanisms involving gene regulation, signal transduction, and other biochemical pathways. In *Catharanthus roseus*, magnesium stress can induce either upregulation or downregulation of transcription factors, such as *ORCA3*, thereby modulating the biosynthesis of alkaloids, including vinblastine [24]. Furthermore, treatment with Ca^2+^ and salicylic acid (SA) significantly enhanced the accumulation of phenolic compounds and flavonoids in *Amaranthus tricolor*, effectively mitigating the adverse impacts of salt stress [25]. In our 2016–2018 trials, six potassium application levels were tested on two *F. thunbergii* cultivars. The results revealed that optimal potassium application increased the bulb steroidal alkaloid content by 2.1–26.6% and yield by 6.4–23.8% [26,27], underscoring its multifaceted regulatory potential. Combined shading and potassium application improved rhizosphere microbial diversity, yield stability, and phytochemical quality, highlighting their synergistic benefits under stress conditions. Therefore, applying potassium under shading can mitigate the shade-induced *F. thunbergii* bulb yield loss.

Steroidal alkaloids, the principal bioactive constituents in *F. thunbergii* bulbs, are critical secondary metabolites with demonstrated health-promoting properties [27]. The 2020 edition of the Chinese Pharmacopoeia specifies peimine and peiminine as the quantitative markers for *F. thunbergii*, mandating a minimum combined content of 0.080% in dried bulbs. Elucidating the biosynthetic pathways of these steroidal alkaloids through biotechnology and synthetic biology is pivotal for the scalable production of bioactive compounds. The cytoplasmic mevalonic acid (MVA) and plastidial methylerythritol phosphate (MEP) pathways are two key biosynthetic pathways that regulate the production of secondary metabolites in medicinal plants during their growth and development. The MVA pathway, comprising six enzymatic steps catalyzed by crucial enzymes, such as 3-hydroxy-3-methylglutaryl coenzyme A reductase (HMGR), 3-hydroxy-3-methylglutaryl-CoA synthase (HMGS), and mevalonate kinase (MK), provides precursor compounds for terpenoids, sterols, and phytohormones [28,29]. The MEP pathway comprises seven enzymatic reactions, initiating with the condensation of pyruvate and glyceraldehyde-3-phosphate by 1-deoxy-D-xylulose-5-phosphate synthase to form 1-deoxy-D-xylulose-5-phosphate. Subsequent steps involve 1-deoxy-D-xylulose-5-phosphate reductoisomerase (DXR), 2-C-methyl-D-erythritol 4-phosphate cytidylyltransferase (MCT), 4-diphosphocytidyl-2-C-methyl-D-erythritol kinase (CMK), and 1-hydroxy-2-methyl-2-butenyl-4-diphosphate synthase (HDS), ultimately generating the universal precursors isopentenyl diphosphate (IPP) and dimethylallyl diphosphate (DMAPP) [30]. These precursors are condensed by farnesyl pyrophosphate synthase (FPS) to generate farnesyl pyrophosphate (FPP), a critical substrate for steroidal alkaloid biosynthesis. As a nodal enzyme, FPS integrates the flux from both pathways, and its gene has been cloned and functionally characterized in *Euphorbia pekinensis* [31], *Rosa rugosa* [32], and *Centella asiatica* [33], with tissue-specific and developmental-stage expression patterns elucidating its regulatory roles. For instance, FPS activity in *Arabidopsis thaliana* modulates isoprenoid precursor accumulation in transgenic seeds [34], while the *Saccharomyces cerevisiae* ERG20 gene (ortholog of FPS) enhances terpenoid synthesis by optimizing intermediate flux [35].

Environmental factors significantly influence steroidal alkaloid accumulation in *F. thunbergii* bulb, as evidenced by studies showing that biocontrol agents enhance steroidal alkaloid yield via modulation of amino acid synthesis and oxidative phosphorylation [36]. Key enzymes, including cytochrome P450 and acetyl-CoA C-acetyltransferase, have been identified as rate-limiting catalysts in these pathways. Intercropping systems, such as *Fritillaria hupehensis* with *Magnolia officinalis*, amplify steroidal alkaloid biosynthesis by regulating phytohormone signaling, oxidative phosphorylation, and cytochrome P450 activity, alongside transcription factors (TFs) such as those of *AP2/ERF* and *WRKY* [37]. Genome-wide analyses have further revealed conserved TFs (e.g., *bHLH* and *WRKY* TFs) that orchestrate alkaloid biosynthesis across species. For instance, *bHLH* and *WRKY* TFs regulate bioactive compound accumulation in *Coptis chinensis* [38], while *AP2/ERF* TFs are central to alkaloid pathway regulation in *Arabidopsis thaliana* [39]. These findings underscore the evolutionary conservation of transcriptional networks governing steroidal alkaloid production, offering targets for metabolic engineering in *F. thunbergii* bulbs.

In recent years, non-standardized cultivation techniques and excessive pursuit of yield enhancement have led to frequent cases of substandard steroidal alkaloid content in *F. thunbergii*. A previous study has shown that the combined factors of shading and potassium application can significantly increase the steroidal alkaloid content in *F. thunbergii* compared to single-factor treatments. In this study, we combined potassium application and shading conditions to explore the molecular mechanisms underlying their synergistic enhancement of steroidal alkaloid biosynthesis in *F. thunbergii* bulbs. We comprehensively analyzed the influence of this interaction on the steroidal alkaloid composition in bulb tissues by employing ultra-high-performance liquid chromatography combined with Fourier-transform mass spectrometry (UHPLC-Q Exactive HF-X). Furthermore, we integrated transcriptome analysis to pinpoint key genes and metabolic pathways regulating steroidal alkaloid accumulation. Additionally, we identified some core structural genes and TFs playing important roles in steroidal alkaloid accumulation through Weighted Gene Co-Expression Network Analysis and studied their coordinated regulatory interactions. Our findings reveal environmental signal integration strategies to optimize the content of specific metabolites, thereby promoting the study of medicinal plant stress physiology.

## 2. Materials and Methods

### 2.1. Materials and Experimental Layout

One-year-old *F. thunbergii* Miq. rhizomes used in the experiment were purchased from Pan’an City, Zhejiang Province, and authenticated by Professor Shuili Zhang from Zhejiang University of Traditional Chinese Medicine as *F. thunbergii* Miq. Field experiments were conducted in Zhuji City, Shaoxing, Zhejiang Province (29°42′40.4″ N, 120°14′31.1″ E). Rhizomes were planted in October 2022 and emerged in February 2023. The experiment comprised 8 treatments: control (CK; no shading or potassium application), K1–K3 (11.2, 22.5, and 45 kg K_2_O/hm^2^, respectively), S (50% shading; Figure S1), and K1S–K3S (combined 50% shading and potassium treatments equivalent to those of K1–K3, respectively). The experiment consisted of the use of potassium fertilizer while bulbs were growing quickly. Two applications of 50% + 50% potassium fertilizer (sulphuric acid potassium fertilizer) were conducted at 20 and 45 days following emergence. A shading net with a 50% shading degree was selected. With a plot size of 1.2 m × 10 m and a planting density of 15 cm × 15 cm in the experimental field, the study used a randomized full-block design with three repeated trials. Throughout the entire cultivation process, the management and application of conventional nitrogen, phosphorus and potassium fertilizers were strictly implemented in accordance with the standardized production protocols established for *F. thunbergii*. For both transcriptomics and metabolomics analyses, 6 biological replicates were established, with 12 uniformly grown plants randomly selected for each replicate to ensure data reliability and reproducibility. Additionally, 6 biological replicates were also established for the analysis of the content and accumulation of active ingredients to ensure the reliability and reproducibility of the data.

### 2.2. Measurement of F. thunbergii Bulb Quality Indices

The high-performance liquid chromatography system used in this study consisted of a 2424 evaporative light-scattering detector and e2695 separation module (ELSD, Waters Corporation, Milford, MA, USA). The liquid chromatography experiments were conducted using a Durashell C18 (L) column (Tianjin Bonnai Ajie Technology Co., Ltd., Tianjin, China). Sample preparation followed the 2020 Chinese Pharmacopoeia Committee guidelines [40]. The powder (2.00 g, sieved through a No. 4 sieve) was weighed into a flask, mixed with 4 mL of concentrated ammonium hydroxide, and soaked for 1 h. Subsequently, 40 mL of chloroform/methanol (4:1) mixture was incorporated, measured, and thoroughly mixed. After 2 h of heating and refluxing at 80 °C in a water bath, the mixture was cooled, reweighed, and topped off with solvent to make up for any weight loss. Following filtration, 10 mL of the filtrate was completely dried in an evaporating dish. The leftover residue was dissolved in methanol, placed in a 2 mL flask, diluted to the appropriate level, and thoroughly mixed. The solution was centrifuged at 12,000× *g* for 5 min, filtered, and moved to a vial for injection purposes. For the reference solution, suitable quantities of peimine and peiminine reference materials were measured, solubilized in methanol, and adjusted to create a mixed solution of 0.2 mg/mL of peimine and 0.15 mg/mL of peiminine.

To estimate the active ingredient content, we used the following calculation method:

(1) $$A=\frac{C\times V}{W}$$

where C indicates the concentration of the sample solution, V is the volume of the constant volume, and W is the weight of the fixed amount of sample.

To estimate the active ingredients accumulated, we used the following calculation method:

(2) B = A × m

where A indicates active ingredient content, and m indicates bulb biomass per plant.

### 2.3. Pharmacology of F. thunbergii Bulb

We selected the bulbs of Zhebei 3, which emerge in 84 days, as the experimental subjects. For pharmacological analyses, ten biological replicates were established to ensure the reliability and reproducibility of the data.

#### 2.3.1. Preparation of Extraction Solution

The crude powder of *F. thunbergii* bulb was boiled and extracted twice in nine times the volume of water. The extraction solutions were combined and concentrated to 0.75, 1.5, and 3.0 g/mL. Fourteen treatments were set up, including CK (blank), K2 (potassium application), S (shading), and K2S (potassium application + shading) as the main treatments, with potassium application consisting of three different concentrations: low, medium, and high. The blank and positive controls were set up as auxiliary treatments.

#### 2.3.2. Ammonia-Induced Cough Mouse Model

SPF-grade ICR mice (50% male, 50% female; 18–22 g) in good health conditions were provided by the Experimental Animal Center of Zhejiang University of Traditional Chinese Medicine (Animal Quality Certificate: SYXK (Hangzhou, China) 2021-0012). The mice were maintained at a temperature of 20–25 °C, 50–70% humidity, 150–200 Lux of light intensity, 12 h light/dark cycle, and <50 dB of noise. Animals were randomly assigned to five groups. The treatment groups received oral administration of *F. thunbergii* bulb aqueous extract at doses of 0.75, 1.5, and 3.0 g/kg, while the positive control group was administered Nin Jiom Pei Pa Koa (a honey-infused loquat syrup containing *Fritillaria cirrhosa*) at 25 mL/kg. The blank group was given an equal volume of physiological saline and treated for 7 d. On the 6th day of administration, mice were subjected to fasting (both food and water) for 12 h. On the 7th day, after 1 h of administration, 12.5% ammonia water was sprayed using an ultrasonic atomizer, and data were recorded.

#### 2.3.3. Tracheal Phenol Red Sputum Excretion Experiment

Five groups of animals were randomly selected. The blank group received an equivalent volume of physiological saline. All mice were treated for 7 days, and on the 6th day after administration, they were fasted (both food and water) for 12 h. The positive group was only administered a dose of 30 mg/kg Ambroxol Hydrochloride by gavage on the 7th day. One hour after administration on the 7th day, mice were intraperitoneally injected with 0.5 mL of 2.5% phenol red. After 30 min, mice were sacrificed, their trachea was separated, and trachea were soaked overnight in solution A. The next day, absorbance was measured at 558 nm.

#### 2.3.4. Xylene-Induced Ear Swelling Experiment

Animals were randomly assigned to five groups. The blank group was given an equal volume of physiological saline. All mice were treated for 7 days, and on the 6th day after administration, they were fasted (both food and water) for 12 h. The positive control group was only administered a dose of 10 mg/kg Dexamethasone Sodium Phosphate Injection by gavage on the 7th day. A total of 45 min after administration on the 7th day, 0.1 mL of xylene was applied to the right ear of the mice. After 1.5 h, mice were sacrificed through cervical dislocation, both ears were collected, and a 9 mm punch was used to cut round pieces from both ears, after which they were weighed on a one-ten-thousandth scale.

### 2.4. Metabolome Analysis by Using UHPLC-Q Exactive HF-X

A 50 mg solid sample of *F. thunbergii* bulb was placed into a 2 mL filter tube. Subsequently, 400 μL of an extraction solution was employed to extract the metabolites from the solid sample. This solution was created by mixing methanol and water in a ratio of 4:1 (*v*/*v*). It had 0.02 mg/mL of l-2-chlorophenylalanine as an internal reference. Winbio-96c high-throughput tissue homogenizer (Shanghai Wanbai Biotechnology Co., Ltd., Shanghai, China) was used to homogenize the bulb samples for 6 min at room temperature (−10 °C) at a homogenization frequency of 50 Hz. After that, a 300W-10L SBL-10DT Ultrasonic Cleaner manufactured (Ningbo Xinshi Biotechnology Co., Ltd., Ningbo, China) was used to perform ultrasonic extraction for 40 min at 5 °C and 40 kHz. The samples were centrifuged for 30 min at 4 °C and 13,000 rpm after being stored at −40 °C for 35 min. The resulting supernatant was meticulously gathered and then moved to injection vials in order to get ready for LC-MS/MS analysis. Quality control (QC) samples were made by combining equal amounts of metabolites from each sample. During analysis, a QC sample was inserted every five to fifteen samples to verify the process’s reproducibility.

The LC-MS/MS analysis of the drug was conducted by Majorbio Bio-Pharm Technology Co., Ltd. (Shanghai, China) utilizing a Thermo UHPLC-Q Exactive HF-X system with an ACQUITY HSS T3 column (100 mm × 2.1 mm i.d., 1.8 μm; Waters, Milford, MA, USA). Mobile phase A was composed of 0.1% (*v*/*v*) formic acid, 5% (*v*/*v*) acetonitrile, and 95% (*v*/*v*) water. Mobile phase B consisted of 47.5% (*v*/*v*) acetonitrile, 47.5% (*v*/*v*) isopropanol, 5% (*v*/*v*) water, and 0.1% (*v*/*v*) formic acid. The chromatographic column’s temperature was consistently kept at 40 °C, and the flow rate was accurately controlled at 0.40 mL/min. 

The mass spectrometry data acquisition was conducted using the Thermo UHPLC-Q Exactive HF-X Mass Spectrometer (Thermo Fisher Scientific, Waltham, MA, USA), which features an electrospray ionization (ESI) source capable of operating in both positive and negative modes. The precise parameter configurations are as follows: The sheath gas flow rate was established at 50 arb, while the auxiliary gas flow rate was determined to be 13 arb. The normalized collision energy was established at 20–40–60 V in a rolling manner for MS/MS analysis. The ion spray voltage fluctuation (ISVF) parameter was established at −3500 V in negative mode and 3500 V in positive mode. The MS/MS resolution was established at 7500, while the entire MS resolution was set at 60,000. The data were obtained using the Data Dependent Acquisition (DDA) method. The mass detection range was established at 70–1050 *m*/*z*, comprehensively encompassing the mass spectrum of the target molecule.

Raw LC/MS data underwent preprocessing, utilizing Progenesis QI software (v 3.0) from Waters Corporation, Milford, CT, USA. The data matrix was optimized by eliminating redundancy and aggregating peaks, discarding any identified false positive peaks, including noise, column bleed, derivatized reagent peaks, and internal standard peaks. This resulted in a data matrix containing the mass-to-charge ratio (*m*/*z*), retention duration, and peak intensity. An internal Majorbio database and open metabolic databases such as HMDB (http://www.hmdb.ca/, accessed on 20 April 2023) and Metlin (https://metlin.scripps.edu/, accessed on 22 April 2023) were compared with the MS spectra. For further analysis, the produced data matrix was uploaded to the Majorbio Cloud Platform (cloud.majorbio.com, accessed on 22 April 2023). At least 80% of the metabolic characteristics included in every sample set were preserved by preprocessing the data matrix. Each metabolic signature was normalized to the aggregate for samples whose metabolite levels fell below the lower limit of quantification. To reduce inaccuracies, peak intensities from mass spectrometry were normalized to create a normalized data matrix. In order to make the final data grid suitable for further study, variables from QC samples with a relative standard deviation (RSD) more than 30% were also removed and converted to a log10 format. In order to perform principal component analysis (PCA) and partial least squares discriminant analysis (PLS-DA), the data matrix was preprocessed in R using the ropls package (Version 1.6.2). Metabolites were classified as significantly differentiable if their variable importance in projection (VIP) values exceeded one. Metabolic pathways for these metabolites were annotated utilizing the Kyoto Encyclopedia of Genes and Genomes (KEGG) database (https://www.kegg.jp/kegg/pathway.html, accessed on 25 April 2023). Fisher’s exact test was employed to identify the biological pathways most relevant to the experimental treatment, and pathway enrichment analysis was conducted utilizing the Python scipy.stats module (v 1.7.0).

### 2.5. RNA Isolation and Transcriptomic Analysis

In this work, the TRIzol reagent (Invitrogen, Waltham, MA, USA) was used to extract the total RNA. The concentration of the sample RNA was determined by NanoDrop spectrophotometer (Thermo Fisher Scientific, Waltham, MA, USA). The Chinese company Meiji Biomedical Technology Co., Ltd (Shanghai Meiji Biomedical Technology Co., Ltd., Shanghai, China). performed the sample testing and sequencing. The unprocessed data and analytical figures on sample sequencing can be accessed through the NCBI website. The initial dataset of short reads was meticulously sourced from the NCBI, encompassing login numbers SRR31178335 through SRR31178358.

Fastp was used to handle raw paired-end reads for quality checking and trimming [41] using the default settings. Trinity was subsequently employed to conduct de novo assembly on the clear data [42]. The assembled sequences were processed for filtering using CD-HIT [43] and TransRate [44], and they were assessed using Benchmarking Universal Single-Copy Orthologs [45]. The transcripts were searched against the NCBI NR, COG, and KEGG databases using Diamond to identify proteins with the highest sequence similarity, aiming to retrieve functional annotations (E-value cutoff < 1.0 × 10^−5^). BLAST (v 2.2.9) was also used [46] to compare the single genes from six databases to obtain annotations. Blast2GO and NR annotations were used to obtain GO annotations [47], and InterProScan5 was utilized for obtaining InterPro annotations [47]. Bowtie2 was utilized to map clean readings to a single gene [48]. Gene expression levels were then determined, and differentially expressed genes (DEGs) were identified by means of PossionDis, with |Fold Change| ≥ 2.00 and false discovery rate (FDR) ≤ 0.001. DEGs were classified, and the functional enrichment analysis pathways was carried out by using phyper to calculate the FDR for each *p*-value further. Significant enrichment refers to an FDR value below or equal to 0.001. Hierarchical cluster analysis (HCA) and heat map generation were performed on the data obtained in the R (version 4.4.2). Before comparing the data, log2 conversion was performed on the gene expression data, followed by quantile normalization.

### 2.6. Weighted Gene Co-Expression Network Analysis (WGCNA)

The WGCNA package in R (version 4.4.2) was employed for constructing the co-expression network. The fragments per kilobase million value of log_2_ was converted, and any value less than 1 was considered to be 0. Six thousand genes were employed for WGCNA analysis in this experiment. Default parameter settings (power 8, TOM type unsigned, minimum module size 30, merged cutting height 0.25) were obtained utilizing the automatic network construction module. An undirected weighted network was subsequently inferred. In this network, we defined modules as clusters that used topological coverage matrices to infer gene clusters with similar expression patterns. The network was visualized using Cytoscape v.3.10.2.

### 2.7. Reverse Transcription Quantitative Real-Time Polymerase Chain Reaction Analysis (qRT-PCR) Analysis

We conducted SYBR Green RT-PCR experiments to validate all selected genes. The primers designed for the experiment are presented in Table S1. The 18s rRNA gene served as the pivotal internal control. The final volume of the reaction for the qRT-PCR amplification of target gene transcripts was 10 µL. The reaction mixture was composed of 1.5 µL of cDNA, 0.6 µL of forward primer, and 0.6 µL of reverse primer. It was further enhanced by adding 5 µL of SYBR qPCR Mix and 2.3 µL of sterile water (Vazyme, Nanjing, China). The reaction steps were as follows: holding stage: 95 °C for 30 s; cycling stage: 95 °C for 10 s, 58 °C for 30 s, and 40 cycles; and melt curve stage: 95 °C for 20 s, 58 °C for 60 s, 95 °C for 30 s and 60 °C for 15 s. A melting curve was generated to ensure primer specificity. The experimental reactions were conducted using a monochrome real-time PCR detection system (Bio-Rad, Hercules, CA, USA). The results were collated and computed by normalizing the average threshold cycle (Ct) values of all experiments to the Ct values of the controls. All qRT-PCRs were conducted three times. The fold change in relative expression levels was computed using the 2^−∆∆CT^ method [49].

### 2.8. Localization and Yeast Two-Hybrid Assay

*FtFPS* and the hub TF in the co-expression network were further investigated. Total RNA was converted into cDNA through reverse transcription (HiScript II Q RT SuperMix for qPCR(+gDNA wiper), Vazyme, Nanjing, China), using the template for cloning the full length of *FtFPS* and the hub TF. *FtFPS* was obtained using the 2 × Fast pfus PCR Moster Mix (Servicebio, Wuhan, China) and subsequently cloned into the PCAMBIA1305 vector. The GV3101 strain carrying the constructed plasmid or an empty control vector was used to transform tobacco leaves. Following 48 h of incubation, the fluorescence signals were observed under a confocal microscope (Zeiss, Oberkochen, Germany). Additionally, the pGBKT7:*FtFPS* plasmid was introduced in yeast cells, which were cultured on selective medium for 2 days.

To examine the protein–protein interaction between *FtFPS* and TF, the yeast strain AH109 was cultivated on YPAD plates. The TF cDNA sequence was cloned into the pGADT7 vector, which has an activation domain, and full-length *FtFPS* was fused with the GAL4 DNA-binding domain in the pGBKT7 vector. After plating the transformed strains on SD-Leu-Trp medium, they were cultured for 2 days at 28 °C. Individual colonies were then serially diluted and spotted onto SD-His/Leu/Trp selective medium for additional examination.

### 2.9. Enzymatic Activity of FtFPS

The recombinant plasmid pET32a-*FtFPPS* was created and introduced into *Escherichia coli* BL21 (DE3). The recombinant *E. coli* was cultured in Luria–Bertani medium supplemented with 0.1 mM isopropyl β-D-1-thiogalactopyranoside to stimulate protein expression (IPTG). After incubation at 25 °C for 24 h, the crude enzyme solution was recovered via ultrasonic disruption for further tests. Protein samples that had undergone sodium dodecyl sulfate-polyacrylamide gel electrophoresis were then stained with Coomassie Brilliant Blue G250 (Amyjet Technology Co., Ltd., Wuhan, China).

With 100 μL of crude enzyme solution and 100 μL of Phosphate-Buffered Saline containing 10 mM MgCl_2_, 40 mM dithiothreitol, 50 μmol/L IPP, and 200 μmol/L DMAPP, the FtFPPS enzymatic reaction system had a total volume of 200 μL. The reaction was conducted at a pH of 7.2 and 37 °C for 2 h. Subsequently, double the volume of methanol was added, and the mixture was shaken to terminate the reaction. After centrifugation at 6000 rpm for protein precipitation removal, the supernatant was analyzed through Gas Chromatography–Mass Spectrometry using Agilent DB-5MS capillary columns (Agilent Technologies, California, USA) (0.25 mm × 30 m, 0.25 μm film thickness) and 1 mL/min of helium as the carrier gas at a steady flow rate. The temperature program was set up as follows: 60 °C for 2 min, 210 °C (increasing the temperature at a rate of 30 °C/min), 250 °C (increasing the temperature at a rate of 4 °C/min), and 300 °C (increasing the temperature at a rate of 40 °C/min) for 5 min. The temperature was maintained at 300 °C for the injector, 280 °C for the ion source, and 280 °C for the transfer line. At 70 eV, electron ionization conditions were applied to a 3 μL sample in splitless mode. The mass scanning range was *m*/*z* 50–500, with a solvent delay of 3 min. Data acquisition and spectral interpretation were conducted using Xcalibur software (v 4.7) coupled with the NIST14 mass spectral library (v NIST14).

### 2.10. Statistical Analysis

The statistical analysis was conducted using SPSS (version 24). The significance of differences (*p* < 0.05) was assessed using one-way analysis of variance. The heat map for this experiment was created using TBtools software (version 2.119).

## 3. Results

### 3.1. Effects of Shading, Potassium Application, and Their Coupling on the Content and Accumulation of Active Ingredients in F. thunbergii Bulbs

**Figure 1:**
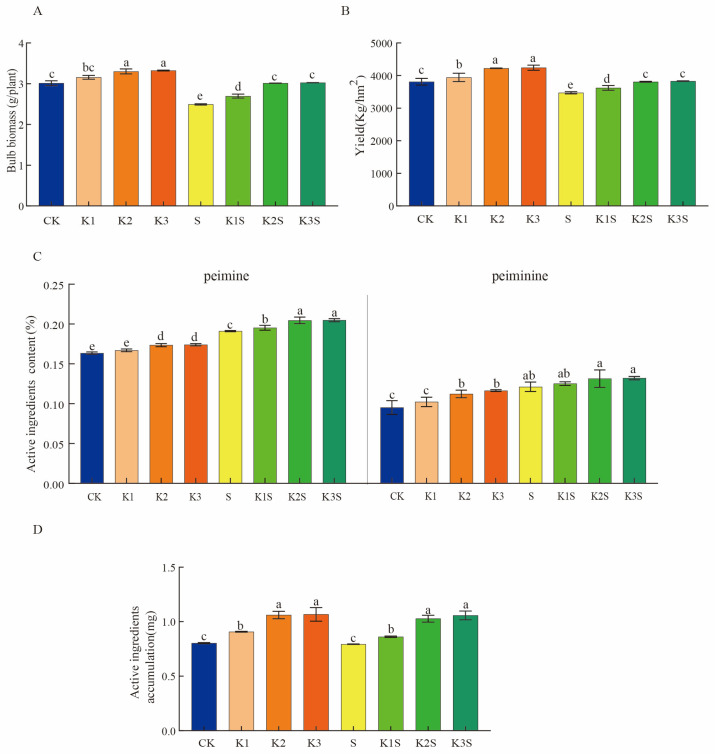
The effects of shading, potassium application, and their coupled treatments on the bulbs of *F. thunbergii*. (**A**) Biomass of individual bulbs of *F. thunbergii* under different treatments. (**B**) The bulb yield of *F. thunbergii* under different treatments. (**C**) Active ingredients’ content in bulbs under different treatments. On the left side of the picture is the content of peimine, and on the right side of the picture is the content of peiminine. (**D**) Active ingredients’ accumulation in bulbs under different treatments. The content of peimine and peiminine were quantified and subsequently summed. The resultant sum was multiplied by the bulb biomass per plant to obtain the overall accumulation of active ingredients. Different lowercase letters indicate statistically significant differences (*p*-value < 0.05) between the treatments.

Compared to the CK group, the S group showed a significant 17.24% decrease in bulb biomass per plant. In the K1–K3 groups, bulb biomass per plant increased in a dose-dependent manner (K1, 4.86%; K2, 9.63%; and K3, 10.33%) compared to that in the CK group. However, there was no significant increase in bulb biomass between K3 and K2. Under shading conditions, the bulb biomass per plant increased with increasing potassium dose application (K1S, K2S, and K3S groups showed increases of 8.21%, 20.93%, and 21.43%, respectively) compared to that in the S group. Notably, the bulb biomass in the K3S group did not significantly differ from that in the K2S group (Figure 1A). The bulb biomass in the K2S and K3S groups was comparable to that in the CK group, with no significant reduction observed. After analyzing the yield data, we found that the trend of differences between each treatment group was consistent with that of single-plant bulb biomass (Figure 1B). These findings indicate that potassium application following shading can compensate for the shade-induced biomass and yield losses in *F. thunbergii* bulb.

According to the Chinese Pharmacopoeia, peimine and peiminine are the primary bioactive steroidal alkaloids in *F. thunbergii* bulb. Analysis of the peimine content demonstrated a significant 16.91% increase in S compared to that in CK. Under normal light, potassium application enhanced peimine accumulation in a dose-dependent manner, with K1, K2, and K3 showing increases of 2.14%, 6.15%, and 6.43%, respectively, compared to CK. Once more, K3 and K2 showed no significant difference. Under shading, the peimine content in the K1S–K3S groups surpassed that in the S group by 2.14%, 7.07%, and 7.15%, respectively, with K3S and K2S showing no significant difference (Figure 1C). Peiminine accumulation followed an identical trend to that of peimine. The K1S–K3S groups exhibited the highest concentrations of peimine and peiminine, followed sequentially by the S, K1–K3, and CK groups. In every paired comparison, significant statistical differences (*p* < 0.05) were found.

The accumulation of bioactive compounds in bulbs was calculated based on the biomass and active ingredient content. Compared to CK, S exhibited a 1.23% reduction in total bioactive compound accumulation. Potassium treatments (K1–K3) under normal light increased this accumulation by 12.98%, 32.08%, and 32.86%, respectively, with K3 and K2 presenting statistically similar results. Under shading, the K1S–K3S groups showed 8.59%, 29.54%, and 33.36% increases in active ingredient accumulation, respectively, compared to the S group; the K3S and K2S groups did not statistically differ. To some extent, treatment with K2S mitigated the reduction in bulb yield caused by treatment S (Figure 1D). Importantly, no significant difference in bulb biomass, yield, active ingredient content, or active ingredient accumulation was observed between K2 and K3 or between K2S and K3S, suggesting that the K2 application rate is the optimal potassium dose for *F. thunbergii* bulb cultivation.

### 3.2. Effects of Shading, Potassium Application, and Their Coupling on the Pharmacology of Active Ingredients in F. thunbergii Bulbs

**Figure 2:**
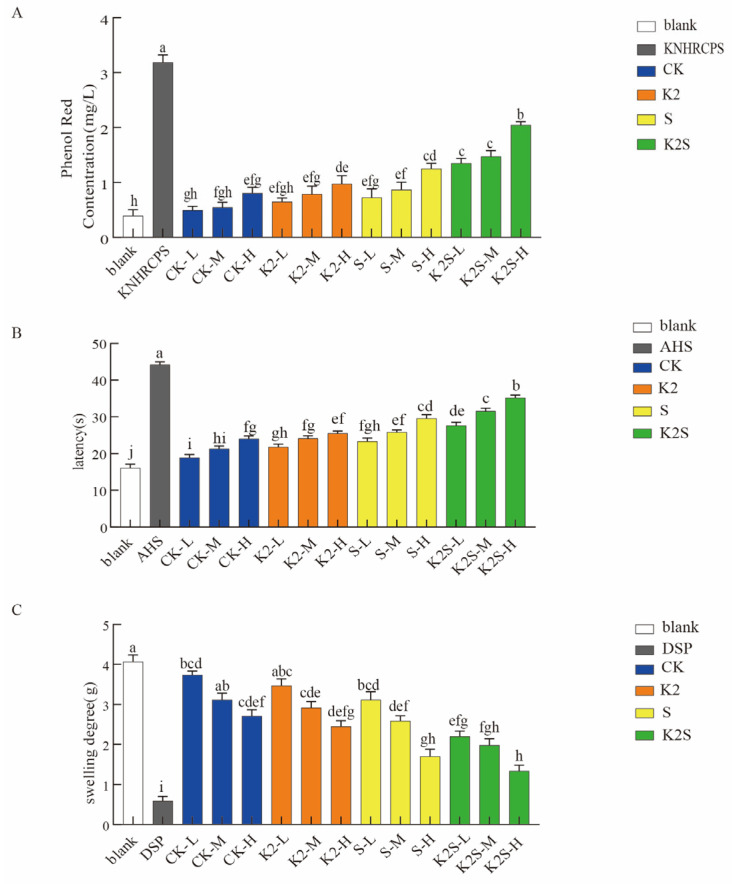
The effects of shading, potassium application, and their coupling treatments on the pharmacological efficacy of *F. thunbergii* bulbs. (**A**) Anti-inflammation experiment. (**B**) Phlegm-resolving experiment. (**C**) Cough test. Different lowercase letters indicate statistically significant differences (*p*-value < 0.05) between the treatments.

To further investigate the pharmacodynamics differences of bioactive compounds in *F. thunbergii* bulbs under four treatments (CK, K2, S, and K2S), three murine pharmacological models were employed: expectorant (phenol red secretion), antitussive (ammonia-induced cough), and anti-inflammatory (xylene-induced ear swelling) activity models. The results showed significant differences in drug efficacy among different treatments, as demonstrated in Figure 2. In contrast to the CK treatment, all other treatments prolonged cough latency. Compared to the blank group, the AHS (positive group), CK, K2, S, and K2S groups had extended latency periods of 174.53%, 19.39–49.07%, 35.40–58.39%, 44.72–83.35%, and 72.05–118.63%, respectively (Figure 2A). Moreover, compared to the blank group, the KNHRCPS (positive group), CK, K2, S, and K2S groups presented increased secretion by 87.77%, 21.42–51.61%, 39.81–59.98%, 46.24–68.84%, and 70.04–80.87%, respectively (Figure 2B). When compared to the blank group, the different treatment groups showed a reduced degree of ear swelling: the DSP (positive group), CK3, K3, S3, and K3S groups showed a swelling inhibition rate of 85.50%, 8.11–33.42%, 14.74–39.80%, 24.08–58.23%, and 49.95–67.08%, respectively. Overall, K2S showed the most significant effect, followed by S and K2 (Figure 2C). Furthermore, K2S demonstrated superior antitussive, expectorant, and anti-inflammatory activities compared to those of S, K2, and CK. This is in line with the findings on the active ingredient content of *F. thunbergii*, indicating that compared to K2 and S alone, the combined K2S treatment enhances the pharmacological effects of *F. thunbergii* bulbs more effectively.

### 3.3. Metabolomic Profiles in Response to Shading, K Application, and Their Coupling Treatments

The compositional differences in metabolites in *F. Thunbergii* bulbs under the K2, S, and K2S groups were analyzed using LC-MS/MS. As is well known, QC samples are helpful in assessing the dependability of metabolomic studies. The peak curves’ substantial overlap indicated the signal’s robustness and consistent outcomes (Figure S2). Model PCA provides a fundamental understanding of sample variability. In the positive ion mode, PC1 and PC2 provided 36.10% and 13.50% of the overall variance, while in the negative ion mode, they contributed 49.60% and 11.40%, respectively (Figure S3). Additionally, the system was dependable and stable. Because each therapy was localized in a distinct area, there were notable variations in the metabolites between the groups as well (Figure S3). In order to guarantee good uniformity, we also conducted correlation analysis between the metabolites in 24 samples. The association in the heat map confirms that the six duplicate samples in each treatment group tend to be grouped (Figure S4). Figure S5 illustrates that the predominant metabolites included lipids and lipid-like molecules, organic acids and their derivatives, organic oxygen compounds, benzenoids, organoheterocyclic compounds, alkaloids and their derivatives, and organic nitrogen compounds. A fair distinction between the various treatment groups was demonstrated by the PLS-DA (Figure S6). The PLS-DA model’s validation results showed that in both the positive and negative ion modes, the R^2^ values were higher than the Q^2^ values. The corresponding intercepts of the Q^2^ regression lines with the Y-axis were −0.2074 and −0.3539. This suggests that the model is appropriate for further data analysis, has a strong predictability, and fits the data well (Figure S7).

**Figure 3:**
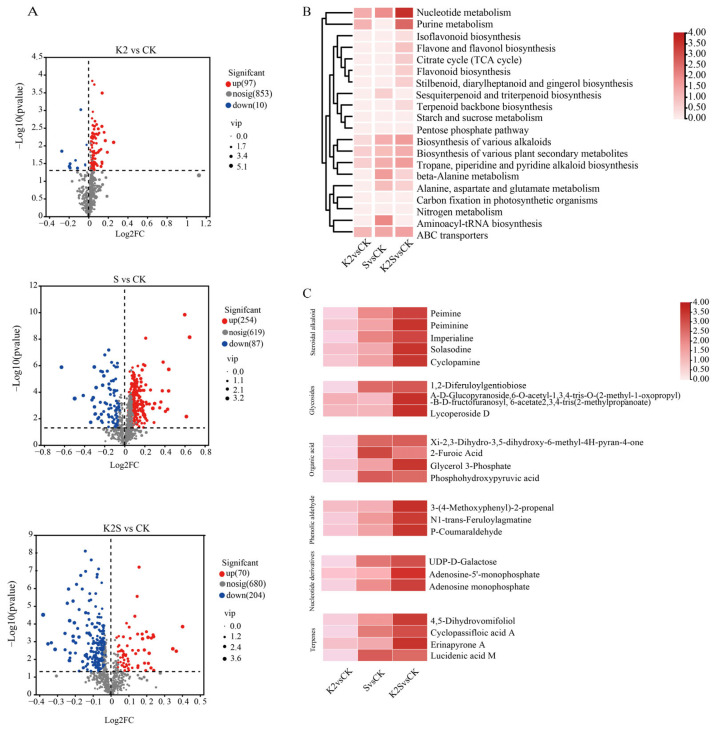
Metabolite quantification and heat map analysis of metabolites KEGG pathway in *F. thunbergii* bulb in response to different conditions. (**A**) Volcano plots of DAMs. (**B**) Heat map illustrating significance of *p*-value for enriched KEGG pathways associated with DAMs in *F. thunbergii* bulbs. (**C**) Heat map illustrating relative abundance of metabolites in *F. thunbergii* bulb. Values of differential metabolite KEGG pathways were normalized and are shown using a color scale.

In total, 959 metabolites were detected in the *F. thunbergii* bulb under the different treatments (Table S2). Differentially accumulated metabolites (DAMs) were identified among metabolites with *p*-value < 0.05. The results are presented in the Venn diagram (Figure S8). In total, 107 DAMs were identified between CK vs. K2. A total of 341 DAMs were identified between CK vs. S. In total, 274 DAMs were identified between CK vs. K2S. Among the 722 DAMs detected (Figure 3A), the number of increased DAMs was 97, 254, and 70 under K2, S, and K2S conditions, respectively, and the number of decreased DAMs was 10, 87, and 204 under K2, S, and K2S conditions, respectively. To cultivate a more profound and intuitive comprehension of the roles played by metabolites exhibiting significant disparities between CK and other treatment conditions, we conducted a KEGG enrichment analysis on the amassed metabolite data. The DAMs were considerably more enriched in the “Biosynthesis of various plant secondary metabolites”, “Biosynthesis of various alkaloids”, “Tropane, piperidine, and pyridine alkaloid biosynthesis”, and “Nucleotide metabolism” in K2, S, and K2S groups. Furthermore, the “beta-Alanine metabolism”, and “Aminoacyl-tRNA biosynthesis” were more enriched between CK and S group, and “isoflavonoid biosynthesis” and “Flavone and flavonol biosynthesis” were more enriched between CK and K2S group (Figure 3B). The HCA results indicated that the metabolite abundance in the K2, S, and K2S groups differed from that in the CK group (Figure 3C). Five steroidal alkaloids were identified, including peimine, imperialine, solasodine, peiminine, and cyclopamine, in the four groups. The Supplementary Information provides a comprehensive presentation of the specifics of these metabolites (Figure S9).

### 3.4. De Novo Assembly and Functional Annotation of F. thunbergii Bulb Under Shading, Potassium Application, and Their Coupling

**Figure 4:**
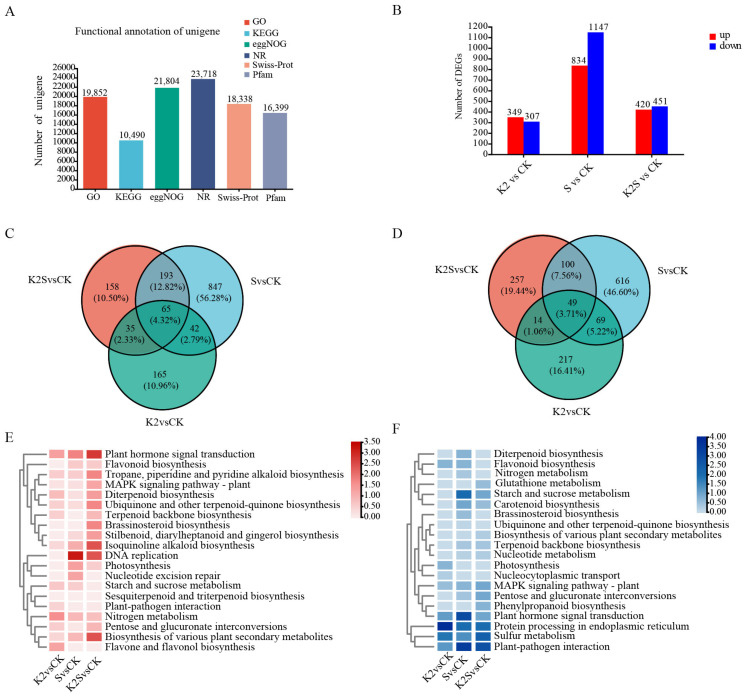
Analysis of *F. thunbergii* bulb transcriptomes in response to different conditions. (**A**) Annotation of unigenes of *F. thunbergii* bulb under different treatments in the GO, KEGG, eggNOG, NR, Swiss-Prot, and Pfarm databases. (**B**) Number of DEGs in *F. thunbergii* bulb under different treatments. Red and blue denote upregulated and downregulated DEGs in *F. thunbergii* bulb, respectively. (**C**,**D**) Venn diagram of upregulated DEGs (**C**) and downregulated DEGs (**D**). (**E**,**F**) Comparative analysis of significantly enriched KEGG pathways in *F. thunbergii* bulb in response to different conditions. Heat map illustrating the significance of *p*-value for enriched KEGG pathways associated with upregulated (**E**) and downregulated (**F**) transcripts in *F. thunbergii* bulb. Upregulated and downregulated pathways were identified based on the criterion *p*-value < 0.05.

Twenty-four RNA Sequencing libraries were sequenced for *F. thunbergii* bulb in response to potassium application and shading conditions, and were designated CK_1-6, K2_1-6, S_1-6, and K2S_1-6. An average of 47.41 million raw reads were sequenced in each library (Table S3). The Q20 and Q30 metrics of the RNA-seq sequences across all samples surpassed 98.33% and 95.16%, respectively, while the GC content proportions were recorded at an impressive 51.26% and 52.46%, respectively. After the assembly process, comprehensive annotations of the constructed unigenes were meticulously conducted utilizing BLAST across six esteemed public databases (GO, NR, KEGG, eggNOG, Pfarm, and Swiss-Prot), and the results were 19,852, 23,718, 10,490, 21,804, 16,399, and 18,338 (Figure 4A). The PCA results showed that the six duplicate samples in the four groups were clustered, and each group could be clearly distinguished (Figure S10).

To enhance the precision in identifying differentially expressed genes (DEGs), we established rigorous selection criteria defined by |Fold Change| ≥ 2.00 and FDR ≤ 0.001 (Figure 4B). A total of 656, 1981, and 871 DEGs were discerned in the *F. thunbergii* bulb under the K2 vs. CK (349 upregulated and 307 downregulated genes), S vs. CK (834 upregulated and 1147 downregulated genes), and K2S vs. CK (420 upregulated and 451 downregulated genes) conditions, respectively. In the comparison between S and CK, the number of downregulated differentially expressed genes (DEGs) was lower than that of upregulated DEGs. The comparison results between K2S and CK were consistent with this observation (Figure 4B). Among the 1505 upregulated DEGs, we identified 65 shared among the K2 vs. CK, S vs. CK, and K2S vs. CK comparisons. Additionally, we detected 165, 847, and 158 specific DEGs in the K2 vs. CK, S vs. CK, and K2S vs. CK comparisons, respectively (Figure 4C). Among the 1322 downregulated DEGs, we identified 49 shared across the K2 vs. CK, S vs. CK, and K2S vs. CK comparisons. Moreover, there were 217, 616, and 257 DEGs uniquely detected in the K2 vs. CK, S vs. CK, and K2S vs. CK analyses, respectively (Figure 4D).

To investigate the biological roles played by these DEGs, a KEGG pathway enrichment analysis was conducted, and key biological processes and major signaling pathways were classified (Figure 4E,F). A total of twenty KEGG pathways were found to be significantly enriched in the *F. thunbergii* bulb. “Tropane, piperidine, and pyridine alkaloid biosynthesis”, “Isoquinoline alkaloid biosynthesis”, “DNA replication”, and “Biosynthesis of various plant secondary metabolites” were all enriched in the K2 vs. CK, S vs. CK, and K2S vs. CK comparisons (Figure 4E). These pathways have a vital role in the biosynthesis of steroidal alkaloid precursors. These pathways were upregulated in the K2, S, and K2S groups compared with the CK group. The transcription level in “Isoquinoline alkaloid biosynthesis” was three-fold higher in the K2S group, one-fold higher in the K2 group, and higher in the S group than in the CK group. Additionally, “flavonoid biosynthesis”, “diterpenoid biosynthesis”, “ubiquinone and another terpenoid-quinone biosynthesis”, and “terpenoid backbone biosynthesis” were more enriched in the K2S group than in the CK group. “DNA replication”, “MAPK signaling pathway-plant”, and “Plant hormone signal transduction”, which are related to signal transduction, were notably upregulated in the K2 vs. CK, S vs. CK, and K2S vs. CK comparisons (Figure 4E). “Photosynthesis” was present in both up- and downregulated pathways. The KEGG analysis findings also showed that among those enriched in DEGs, pathways were significantly downregulated for K2 vs. CK, S vs. CK, and K2S vs. CK, including “Plant hormone signal transduction” and “Sulfur metabolism” (Figure 4F).

### 3.5. Steroidal Alkaloid Biosynthesis in F. thunbergii Bulb Rapidly Reprogrammed by Shading, Potassium Application, and Their Coupling

**Figure 5:**
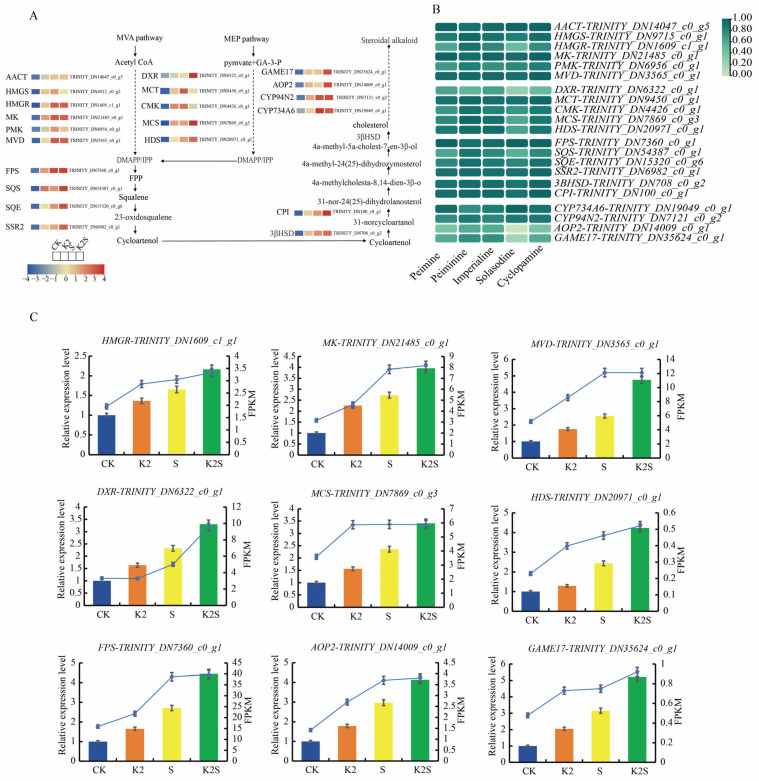
Steroidal alkaloid biosynthesis in *F. thunbergii* bulb in response to different treatments. (**A**) A heat map illustrating gene expression levels. The color scale, ranging from blue to white to red, corresponds to low, medium, and high expression levels, respectively. (**B**) Heat map depicting correlation between fragments per kilobase million (FPKM) values of DEGs and relative abundance of steroidal alkaloids. Pearson’s R-value is provided to indicate the strength of the correlation. (**C**) qRT-PCR and sequencing data of the gene expression in steroidal alkaloid biosynthesis in *F. thunbergii* bulb. The histogram represents outcomes of the qRT-PCR analysis; the line chart depicts sequencing data associated with gene expression.

To screen and determine candidate genes that may be associated with the steroid alkaloid biosynthesis pathway in *F. thunbergii* bulbs, we created a thorough route diagram that included expression heat maps of the structural genes in the bulbs of *F. thunbergii* under various treatments. These genes were anticipated to be crucial to the complex steroidal alkaloid synthesis pathways (Figure 5A). The MVA and MEP signaling pathways represent pivotal mechanisms in the intricate biosynthesis of DMAPP and IPP. These substances are building blocks for the production of steroidal alkaloids, underscoring their significance in metabolic processes [50]. A steroidal alkaloid biosynthesis pathway enriched in 21 DEGs was identified. Upstream of the MVA pathway, the expression of the six transcripts was markedly elevated in the K2, S, and K2S groups compared to that in the CK group (Figure 5A). We observed that *FtHMGR-TRINITY DN1609_c1_g1* (encoding 3-hydroxy-3-methylglutaryl coenzyme A reductase), *FtMK-TRINITY_DN21485_c0_g1* (encoding mevalonate kinase), and *FtMVD-TRINITY_DN3565_c0_g1* (encoding mevalonate diphosphate decarboxylase) were significantly upregulated in the K2, S, and K2S groups. These genes have been documented to contribute significantly to the biosynthesis of DMAPP/IPP via the MVA pathway [51]. Three other transcripts, *FtAACT-TRINITY_DN14047_c0_g5* (encoding acetyl-CoA acetyltransferase), *FtHMGS-TRINITY_DN4912_c0_g2* (encoding 3-hydroxy-3-methylglutaryl-CoA synthase), and *FtPMK-TRINITY_DN6956_c0_g1* (encoding phosphomevalonate kinase), presented higher expression in the K2, S, and K2S groups than in the CK group (Figure 5A). To ensure the accuracy of the transcriptome data, we conducted qRT-PCR to verify the expression of three transcripts (HMGR, MK, and MVD) related to the MVA pathway. The expression patterns of these three transcripts were in accordance with their transcriptomics expression profiles (Figure 5C).

The expression of transcripts recognized as involved in the MEP pathway across numerous plants [52], including *FtDXR-TRINITY_DN6322_c0_g1* (encoding 1-deoxy-D-xylulose-5-phosphate reductoisomerase), *FtMCS-TRINITY_DN7869_c0_g3* (encoding 2-C-methyl-D-erythritol 2,4-cyclodiphosphate synthase), and *FtHDS-TRINITY_DN20971_c0_g1* (encoding 1-hydroxy-2-methyl-2-butenyl-4-diphosphate synthase), was also significantly higher in the K2, S, and K2S groups than in the CK group (Figure 5A). The results of the qRT-PCR analysis confirmed the findings derived from the RNA-seq data (Figure 5C). The expression of two other transcripts, *FtMCT-TRINITY_DN9450_c0_g1* (encoding 2-C-methyl-D-erythritol-4-phosphate cytidylyltransferase) and *FtCMK-TRINITY_DN4426_c0_g1* (encoding 4-diphosphocytidyl-2-C-methyl-D-erythritol kinase), was higher in the S group when compared to that in the CK group (Figure 5A). These findings suggest that the MVA and MEP pathways are significant factors for the biosynthesis of crucial intermediates of steroidal alkaloids in the *F. thunbergii* bulbs, particularly under shading conditions and potassium application.

As a substrate, DMAPP/IPP undergoes a series of chemical reactions through terpenoid skeleton biosynthesis, forming metabolic intermediates such as FPP, squalene, 2,3-oxide squalene, and cycloartenol [53]. Substances, such as FPS, squalene synthase (SQS), squalene epoxidase (SQE), and sterol side chain reductase 2 (SSR2), are engaged in this response process. For DEGs identified under the four treatments, we noted that the expression of the candidate transcripts for these enzymes (*FtFPS-TRINITY_DN7360_c0_g1* encoding FPS; *FtSQS-TRINITY_DN54387_c0_g1* encoding SQS; *FtSQE-TRINITY_DN15320_c0_g6* encoding SQE; and *FtSRR2-TRINITY_DN6982_c0_g1* encoding SSR2) was markedly increased in the K2, S, and K2S groups compared to that in the CK group (Figure 5A). Figure 5C shows that the expression of *FPS* peaked in the K2S group. qRT-PCR was employed to validate the RNA-seq data. Cycloartenol is a key substrate for cholesterol formation and precursor for the synthesis of steroid alkaloids. Based on our analysis, the candidate transcripts, including *Ft3βHSD-TRINITY_DN708_c0_g2* (encoding 3-beta-hydroxysteroid-dehydrogenase 3βHSD) and *FtCPI-TRINITY_DN100_c0_g1* (encoding cyclopropyl sterol isomerase CPI), are related to the conversion of cyclopentanol to cholesterol and showed higher expression in the K2, S, and K2S groups than in the CK group (Figure 5A).

The transformation of cholesterol into a diverse array of steroidal alkaloids encompasses a series of intricate reactions, including oxidation, hydroxylation, glycosylation, and transamination. The candidate transcripts (*FtCYP734A6-TRINITY_DN19049_c0_g1*, encoding cytochrome P450 734A6 CYP734A6; *FtCYP94N2-TRINITY_DN7121_c0_8*, encoding cytochrome P450 94N2 CYP94N2; *FtAOP2-TRINITY_DN14009_c0_g1*, encoding antioxidant protein 2 AOP2; and *FtGAME17-TRINITY_DN35624_c0_g1*, encoding gamma-solanine acetyltransferase 17 GAME17) were determined in the four treatment groups. The expression of these transcripts in the K2, S, and K2S groups was elevated compared to that in the CK group (Figure 5A). These findings confirm that the expression patterns of *AOP2* and *GAME17* consistently align with the respective transcriptome expression profiles (Figure 5C).

Subsequently, the R-values between the 21 DEGs and 5 primary steroidal alkaloids (peimine, peiminine, imperialine, solasodine, and cyclopamine) were calculated (Figure 5B). The transcripts encoding *FtAACT*, *FtMK*, *FtMVD*, *FtFPS*, *FtMCT*, *FtSSR2*, *Ft3βHSD*, and *FtCPI* exhibited a significant positive correlation with steroidal alkaloids such as peimine, peiminine, imperialine, solasodine, and cyclopamine (*p* < 0.05; R > 0.80). These findings suggest that these transcripts are critical in the synthesis of steroid alkaloids. Furthermore, the R-values between the transcripts encoding MK and peimine, imperialine, and cyclopamine were 0.99, 0.97, and 0.99, respectively, suggesting that MK is involved in peimine, imperialine, and cyclopamine biosynthesis. The R-value of the gene encoding FPS and solasodine was 0.97, suggesting that FPS engages in solasodine biosynthesis. Similarly, the R-value for the gene encoding CPI and peiminine was 0.96, indicating that CPI might be involved in the biosynthesis of peiminine (Figure 5B).

### 3.6. Identification of TFs Potentially Involved in the Biosynthesis of Steroidal Alkaloids in F. thunbergii Bulb

**Figure 6:**
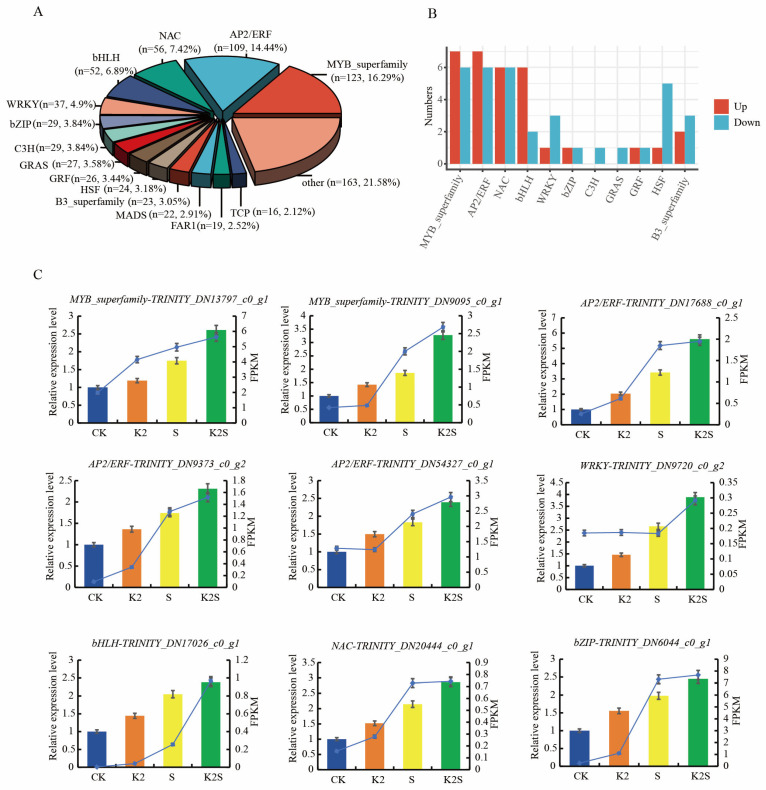
Differential expression analysis of predicted TFs in response to different treatments. (**A**) Type and number of major differential TFs. (**B**) DEGs of predicted TFs in *F. thunbergii* bulb. (**C**) qRT-PCR and sequencing data of the gene expression of TFs in *F. thunbergii* bulb. The histogram illustrates the results of qRT-PCR analysis; the line chart depicts sequencing data related to gene expression.

TFs are essential for regulating plant metabolism and seed germination [54,55]. A comparative analysis was conducted to identify the TFs involved in steroidal alkaloid synthesis in *F. thunbergii* bulbs. In total, 755 RNA-seq transcripts containing TF domains were identified and subsequently classified into 15 TF families, including MYB (123), AP2/ERF (109), NAC (56), bHLH (52), WRKY (37), and bZIP (29) (Figure 6A). Notably, the majority of genes encoding *FtMYB*, *FtAP2/ERF*, *FtNAC*, *FtbHLH*, and *FtbZIP* were upregulated (Figure 6B). Seven *MYB* family members and seven *AP2*/*ERF* genes were upregulated under at least one treatment. Other TFs related to steroidal alkaloid biosynthesis, including *FtWRKY*, *FtbHLH*, and *FtbZIP*, were also identified. Six *NAC* and six *bHLH* genes were upregulated after at least one treatment. One *WRKY* gene was upregulated in at least one treatment. These TF families are closely associated with carrier steroidal alkaloid synthesis.

Two *MYB* superfamily transcripts (*TRINITY_DN13797_c0_g1* and *TRINITY_DN9095_c0_g1*) were identified in the K2 vs. CK, S vs. CK, and K2S vs. CK comparisons through qRT-PCR. The expression of *TRINITY_DN9095_c0_g1* (*MYB_superfamily*) demonstrated a notable increase in the K2S vs. CK comparison (Figure 6C). Furthermore, three *AP2/ERF* transcripts (*TRINITY_DN17688_c0_g1*, *TRINITY_DN9373_c0_g2*, and *TRINITY_DN54327_c0_g1*) were identified in the K2 vs. CK, S vs. CK, and K2S vs. CK comparisons. Among these three *AP2/ERF* transcripts, *TRINITY_DN9373_c0_g2* was upregulated in the K2, S, and K2S groups compared to the CK group (Figure 6C). One *NAC* (*TRINITY_DN20444_c0_g1*), one *bHLH* (*TRINITY_DN17026_c0_g1*), one *WRKY* (*TRINITY_DN9720_c0_g2*), and one *bZIP* (*TRINITY_DN6044_c0_g1*) transcript were detected in the K2 vs. CK, S vs. CK, and K2S vs. CK comparisons through qRT-PCR, and their expression was markedly upregulated in the K2S group compared to the CK group (Figure 6C).

### 3.7. WGCNA of F. thunbergii Bulb in Response to Shading, Potassium Application, and Their Coupling

**Figure 7:**
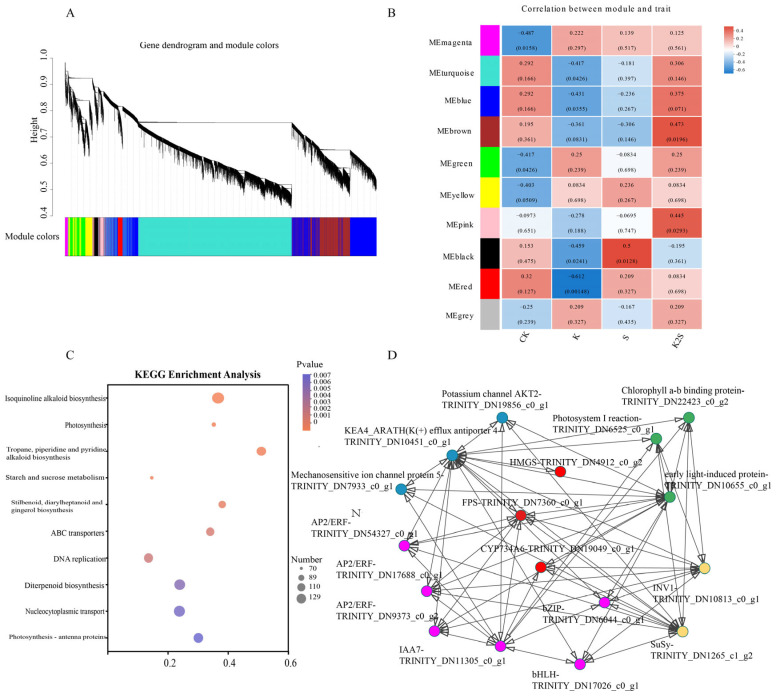
Co-regulated gene expression network of *F. thunbergii* bulb in response to different conditions. (**A**) Module and sample correlation analysis. Hierarchical cluster tree showing co-expression modules identified by WGCNA. Each leaf of the dendrogram represents an individual gene. Each colors represents a module in which the gene expression trends have similar characteristics. (**B**) Module cluster analysis. Each row corresponds to a module. (**C**) KEGG enrichment analysis with all the genes in the pink module. (**D**) Pink module network. Red nodes are related to steroidal alkaloid biosynthesis; green nodes are related to light; blue nodes are related to ion channel protein; yellow nodes are related to starch and sucrose metabolism; pink nodes are related to the transcription factor. Cytoscape visualizes genes with an edge weight greater than 0.2.

To elucidate the pivotal regulatory factors or pathways within the intricate network of the *F. thunbergii* bulb, we used WGCNA to comprehensively analyze co-expression data for all DEGs in response to potassium application and shading conditions (Figure 7A). Figure 7B illustrates the establishment of a ten-module clustering, with samples collected from the four treatments corresponding to each respective module. KEGG enrichment analysis indicated that the differentially expressed genes in the pink module were significantly associated with photosynthesis, tropane, piperidine, and pyridine alkaloid production, ABC transporters, and DNA replication (Figure 7C). In the pink module, three DEGs represented as red nodes, specifically *FtHGMS*, *FtFPS*, and *FtCYP734A6*, were classified into the steroidal alkaloid biosynthesis pathway. Three DEGs shown as blue nodes, namely Potassium channel AKT2, KEA4_ARATH(K(+) efflux antiporter 4), and Mechanosensitive ion channel protein 5, were categorized into the ion channel protein gene. Three DEGs shown as green nodes, namely Photosystem I reaction, Chlorophyll a-b binding protein, and early light-induced protein, were related to light. Six DEGs shown as pink nodes, including *FtAP2/ERF*, *FtbHLH*, and *FtbZIP*, were categorized as TFs. Two DEGs shown as yellow nodes, namely *FtINV1* and *FtSuSy*, were categorized into starch and sucrose metabolism (Figure 7D). In this module, as an important core node gene, *FtFPS* is regulated by potassium channel protein and light response gene, and also interacts with the core TF gene *FtAP2*/*ERF*. DEGs including *FtFPS*, *FtAP2*/*ERF*, *FtbHLH*, and *FtbZIP* were also confirmed through qRT-PCR (Figure 5C and Figure 6C). Table S4 lists DEGs potentially participating in the *F. thunbergii* bulb network including steroidal alkaloid biosynthesis and TFs.

### 3.8. Location and Functional Verification of FtFPS Protein

**Figure 8:**
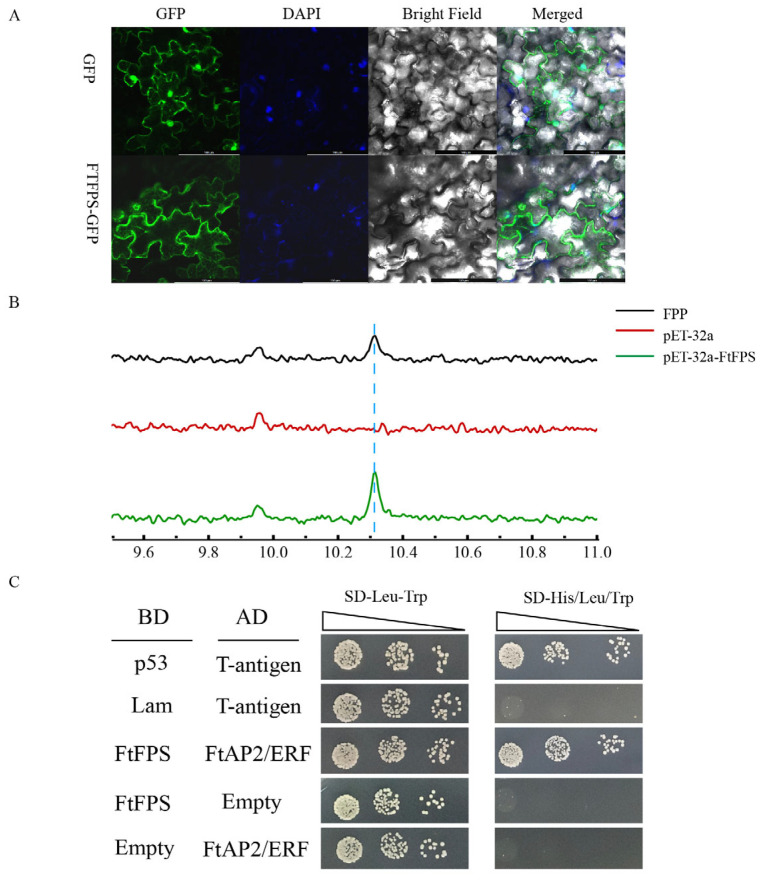
Comprehensive analysis of FtFPS. (**A**) Subcellular location of FtFPS in tobacco. DAPI was used as a nucleus marker. Green represents the GFP fluorescence signal and blue represents the fluorescence signal of the cell nucleus. Scale bars: 100 μm. (**B**) Results of FtFPS catalytic reaction by GC-MS. The blue dotted line represents the peak position of FPP. (**C**) Yeast two-hybrid (Y2H) analysis of FtFPS and FtAP2/ERF. Interaction of FtFPS (fused to BD, *pGBKT7*) with FtAP2/ERF (fused to AD, *pGADT7*) by a Y2H experiment. p53 (*pGBKT7-53*)/T-antigen (*pGADT7-T*) and Lam (*pGBKT7*-Lam)/T-antigen were positive and negative controls, respectively.

FPP is the common precursor for steroidal alkaloid biosynthesis, and is synthesized from IPP and DMAPP by FPS. *FtFPS* (*TRINITY_DN7360_c0 g1*) had an open reading frame sequence of 1056 bp, which encoded a 351 amino acid protein with a calculated molecular weight of 40.06 kDa. FtFPS was further investigated in this study to explore its regulatory role within the network. To understand the subcellular localization of FtFPS, a transient transformation experiment was carried out, and the results indicated that the fluorescent signal was solely detected in the cytoplasm of tobacco leaf cells (Figure 8A). IPTG was then used to produce the FtFPS protein, and it was found that its molecular weight matched the theoretical one (Figure S11). The substrates IPP and DMAPP were catalyzed by the crude enzyme of FtFPS in the reaction system. A novel peak appeared at 10.315 min in comparison to the control, possibly representing putative FPP (Figure 8B). Thus, these findings demonstrate that FtFPS promotes the synthesis of steroidal alkaloids. To further validate the regulatory role of FtFPS, a yeast two-hybrid (Y2H) experiment was conducted to investigate the potential interaction between FtFPS and AP2/ERF (*TRINITY_DN9373_c0_g2*). Yeast cells in both the experimental and positive control groups exhibited normal growth on the screening medium, whereas those in the negative control group did not grow (Figure 8C). Collectively, these results indicate that FtFPS regulates steroidal alkaloid production by augmenting the transcriptional activity of its target FtAP2/ERF.

## 4. Discussion

*F. thunbergii* is a medicinal source owing to its cough-suppressive, anti-inflammatory, and antitumor properties [56]. This medicinal efficacy is mainly attributed to its active ingredients, particularly steroid alkaloids [57]. Recently, wild resources of *F. thunbergii* bulb have been depleted due to overexploitation, and bioengineering may be the most feasible approach to address this issue [58,59,60].

### 4.1. Synergistic Application of Shading and Potassium Exerts Dual Regulatory Effects on Both the Yield Stability and Quality of F. thunbergii Bulbs

In this study, compared to the CK treatment, K1–K3 treatments significantly increased the biomass and yield of *F. thunbergii* bulbs by 4.86–10.33% and 3.84–11.24%, respectively, with this increase being potassium-dose-dependent (Figure 1A,B). This indicates that applying potassium fertilizer within a certain dose range can significantly increase dry matter in medicinal plants [61]. Research has found that applying potassium fertilizer within a certain dose range can significantly increase the yield of *Vigna radiata* (L.) R.Wilczek. [62]. A shaded environment negatively affects plant yield, and a light transmission rate of 5% severely affects the yield of *F. thunbergii* [15]. In our study, the biomass and yield of *F. thunbergii* bulbs in group S were significantly reduced by 17.24% and 8.90%, respectively, supporting the latter. The bulb biomass and yield in the K1S–K3S groups exhibited a gradual increase with the rise in potassium application rates, with increments of 8.21–21.43% and 4.29–10.25%, respectively. Despite observable differences, these increases were not statistically significant and remained slightly lower than those observed in the CK group. The augmentation of bulb biomass and yield with elevated potassium application levels indicates that the synergy of shading and potassium application mitigates the shade-induced decline in these parameters. However, no significant change in bulb biomass or yield was observed between groups K2 and K3, or between groups K2S and K3S, indicating that the medium potassium dose is enough to exert the most beneficial effects (Figure 1A,B).

Figure 1C,D shows the analysis of the active ingredient content and accumulation in *F. thunbergii* bulbs. In comparison with those in the CK group, the bulb active ingredient content and accumulation in groups K1–K3 showed a significant increase. The active ingredient content in the bulbs of group S increased significantly by 20.71%, whereas the active ingredient accumulation decreased by 1.23%. Groups K1S–K3S presented significantly higher active ingredient content and accumulation than groups S (2.64–7.87% and 8.59–33.36%, respectively) and CK (23.90–30.21% and 7.26–31.72%, respectively). Through three in vivo pharmacological experiments (Figure 2), we measured the antitussive, expectorant, and anti-inflammatory effects of *F. thunbergii* bulbs grown under different treatments. Compared to the blank treatment, the K2S treatment induced the strongest antitussive, expectorant, and anti-inflammatory effects, followed by the S, K2, and CK treatments. This is consistent with the active ingredient content of *F. thunbergii* bulbs observed in this study, indicating that the combined K2S treatment enhances the pharmacological effects of *F. thunbergii* bulbs more effectively than the K and S treatments alone.

Considering the abovementioned findings, potassium fertilizer not only affects yield but also directly influences the synthesis, distribution, and accumulation of active ingredients, affecting plant quality [61]. Shading can reduce photoinhibition and optimize photosynthetic efficiency [63]; although it may inhibit yield growth, shading significantly increases the active ingredient content and accumulation, exerting better effects than those triggered by potassium fertilizer application. Under shading conditions, the alcohol-soluble protein content of glutinous corn kernels increases [64]. The simultaneous application of shade and potassium mitigated the shade-induced reduction in *F. thunbergii* bulb production and biomass, while significantly improving the bulb active ingredient content and accumulation.

### 4.2. Untargeted Metabolomics Reveals Steroidal Alkaloid Dynamics in F. thunbergii Bulbs Under Different Treatments

The metabolites in the bulbs of *F. thunbergii* mainly include lipids and lipid-like molecules, organic acids and derivatives, organic oxygen compounds, benzenoids, organoheterocyclic compounds, alkaloids, and derivatives, and organic nitrogen compounds were the most common metabolites. Based on the metabolomics analysis (Figure 3), the primary metabolites in the bulbs of *F. thunbergii* are steroidal alkaloid compounds, with peiminine and peimine as the core pharmacological components [55,65]. The main active ingredients responsible for the heat-clearing, phlegm-resolving, and cough-relieving effects of *F. thunbergii* bulbs are indeed peiminine and peimine [66,67]. This investigation expands upon prior studies on steroidal alkaloids, as it identifies three new steroidal alkaloids with potential therapeutic value: imperialine, solasodine, and cyclopamine. Experimental data show that the relative abundance of imperialine, solasodine, and cyclopamine significantly increased after the K2S, S, and K2 treatments, with the K2S group showing the most significant enrichment, followed by the S and K2 groups. This demonstrates that potassium application and shading are effective agronomic measures to enhance *F. thunbergii* bulb active components. Imperialine, as an efficient expectorant and antitussive drug, is primarily extracted from the bulbs of *Fritillaria cirrhosa* [68]. Solasodine exerts anticancer effects by finely regulating colorectal cancer stem cell characteristics and epithelial–mesenchymal transition processes, with the use of its derivatives considered a novel natural anticancer strategy [69]. Cyclopamine, a naturally occurring steroidal alkaloid, shows broad prospects in tumor-targeted therapy [70]. These compounds collectively constitute the chemical basis for the anti-inflammatory activity of *F. thunbergii* bulbs. The metabolites identified in this study not only complete the chemical profile of steroidal alkaloids in *F. thunbergii* bulbs but also provide a new perspective by elucidating their pharmacological substance basis, holding significant scientific value for further exploration of active components in this medicinal plant.

### 4.3. Transcriptome-Based Analysis of the Steroidal Alkaloid Biosynthesis Pathway in F. thunbergii Bulbs

Steroidal alkaloids constitute a category of intricate natural substances produced via several biological processes. In recent years, a multitude of studies have focused on the biosynthesis pathways of several steroidal alkaloids. In plants, the complex skeletons of terpenoids are primarily synthesized from DMAPP and IPP via the MVA and MEP pathways [3,71]. Our study findings indicate that six genes associated with DMAPP/IPP production via the MVA route were highly expressed in the K2S group (Figure 5). The expression of the MVA pathway-related genes *FtHMGR*, *FtPMK*, *FtMK*, *FtMVD*, *FtAACT* and *FtHMGS* was significantly elevated in the K2S group. Additionally, five genes participating in DMAPP/IPP production via the MEP pathway showed high expression in the K2S group. In accordance with a recent study, we hypothesize that the MVA pathway is the primary mechanism involved in the production of DMAPP/IPP precursor chemicals. When potassium application and shading are combined, the accumulation of DMAPP and IPP intermediates in the bulbs of *F. thunbergii* is enhanced.

FPS produces common precursors for the metabolism of plant sterols and cholesterol. Located at the initial branching point of the MVA pathway, the FPS catalytic product, FPP, acts as a precursor for the synthesis of plant sterols, steroids, terpenes, and other compounds. Plant sterols also contribute to the steroid nucleus structure needed for the synthesis of steroid alkaloids. Consequently, it is inferred that the MVA pathway supplies precursor substances for the synthesis of steroid alkaloids [72,73]. Consequently, the *FtFPS* gene may participate in regulating the biosynthesis of steroid alkaloids in *F. thunbergii*. In this study, the *FtFPS* gene exhibited the highest expression under the K2S treatment, followed by the S, K2, and CK treatments. This suggests that combining shading and potassium application significantly enhances catalysis by FtFPS, increasing the synthesis and accumulation of its product, FPP. Among single-factor treatments, shading had a more pronounced effect on FtFPS catalysis than potassium application. FtSSR2 coordinates the conversion of cycartenol to cycloartanol, a crucial event that demonstrates its function as the starting enzyme in the intricate process of cholesterol biosynthesis [74]. The K2S, S, and K2 groups exhibited noticeably increased expression of *FtFPS* and *FtSSR2*. The identified upregulated key genes are associated with the biosynthesis of the terpene skeleton, suggesting that they regulate steroidal alkaloid biosynthesis by affecting precursor supply. In this study, we successfully identified four promising candidate genes, *FtCYP734A6*, *FtCYP94N2*, *FtAOP2*, and *FtGAME17*, that showed significantly increased expression in the K2S group.

TFs play direct or indirect roles in plant secondary metabolism. Previous studies have elucidated the regulatory functions of several TF families, such as bHLH, AP2/ERF, MYB, WRKY, and bZIP, in alkaloid biosynthesis [75,76]. These TFs are essential for controlling the biosynthesis of alkaloids in *Catharanthus roseus*, *Ophiorrhiza pumila*, and *Nelumbo nucifera* [77,78,79]. In *Fritillaria* species, the AP2/ERF, MYB, NAC, and bHLH families were found to regulate steroidal alkaloid biosynthesis [80,81]. In the present study, we determined TF family members such as AP2/ERF to be involved in steroidal alkaloid synthesis pathways in the bulbs of *F. thunbergii* treated with a combination of potassium and shading (Figure 6). These TFs exhibited high expression in the K2S, S, and K2 groups, and their expression patterns were validated using qRT-PCR. Studies have demonstrated that the *AtAP2*/*ERF* gene family plays a crucial role in steroidal alkaloid biosynthesis [39]. In our experiments, three *AP2*/*ERF* genes showed the significant expression in the K2S group, suggesting their potential dominance in steroidal alkaloid biosynthesis in *F. thunbergii* under combined treatment conditions. The MYB family serves as core regulatory elements in the steroidal alkaloid biosynthesis network [82], with specific metabolic pathways in *Ophiorrhiza pumila* being regulated by them [83]. The mechanism of action of the WRKY family aligns with that described in previous research: overexpression of WRKY in *Coptis japonica* significantly increases the benzylisoquinoline alkaloid (BIA) content [84,85]; furthermore, six WRKY TFs found in *Nelumbo nucifera* control the production of BIA [86], while *NnWRKY70a/b* significantly enhances BIA accumulation by upregulating structural gene expression [87]. According to our research, the K2S group showed considerably higher WRKY transcript expression than the other groups, further supporting the positive regulatory role of this family in *F. thunbergii* steroidal alkaloid biosynthesis. These TFs collectively regulate the steroidal alkaloid biosynthetic pathway in *F. thunbergii* bulbs through both direct and indirect mechanisms.

### 4.4. Co-Expression Module–Transcriptome Association Analysis and Identification of Rate-Limiting Nodes in Steroidal Alkaloid Biosynthesis

This study reveals the gene regulatory network of *F. thunbergii* bulbs under potassium application and shading conditions through WGCNA analysis (Figure 7). DEGs in the pink module are closely related to photosynthesis, steroidal alkaloid biosynthesis, ABC transporters, and DNA replication, indicating that these genes serve essential functions related to the bulb’s adaptation to environmental stress. Potassium, as a key nutritional element for plant growth and development, may influence the activity of genes associated with photosynthesis and steroidal alkaloid biosynthesis by regulating the expression of ion channel proteins and TFs [88], thereby promoting bulb growth and development. Shading conditions may regulate photosynthetic efficiency by affecting light reaction genes, subsequently impacting the physiological metabolism of the bulb [89].

*FtFPS*, a core node gene in the pink module, is regulated by potassium channel proteins and light reaction genes, and interacts with the core TF gene *FtAP2/ERF*. This regulatory relationship indicates that FtFPS occupies a critical position in the gene regulatory network of *F. thunbergii* bulbs. By controlling the expression of genes linked to steroidal alkaloid biosynthesis, FPS may have an impact on the generation of secondary metabolites in bulbs, thus enhancing their stress resistance and quality [90]. Additionally, the interaction between FPS and the TF AP2/ERF may further regulate the expression of other genes, forming a complex regulatory network [91].

Furthermore, we cloned the complete sequence of the *FtFPS* gene in *F. thunbergii* bulbs (Figure 8), revealing its involvement in the biosynthesis and metabolic regulation of steroidal alkaloids in *F. thunbergii* bulbs [90]. The *FtFPS* gene is a key node gene in the MVA and MEP pathways within plants, regulating not only steroid alkaloid synthesis but also the synthesis of terpenoids, plant hormones, and sterols [92]. Overexpression of *BpFPS* significantly increases the triterpene content in *Betula platyphylla* Suk. [93]. Our study confirmed through transient transformation experiments that FtFPS is localized in the cytoplasm, consistent with results from experiments on the *Atractylodes macrocephala FPS* gene [92]. The FtFPS protein was expressed in *E. coli* and was found to catalyze the synthesis of FPP from IPP and DMAPP. AP2/ERF TFs play significant roles in transcriptional regulation across various plants [94], with studies showing that the *AP2/ERF* gene family positively regulates benzylisoquinoline alkaloid synthesis in *C. chinensis* [95]. Herein, we also cloned the *FtAP2/ERF* gene and validated the interaction between FtAP2/ERF and the FtFPS functional gene through Y2H experiments. These findings offer significant insights into the adaptive mechanisms of *F. thunbergii* bulbs under different environmental conditions, and theoretical support for genetic improvement and cultivation management of *F. thunbergi*.

## 5. Conclusions

**Figure 9:**
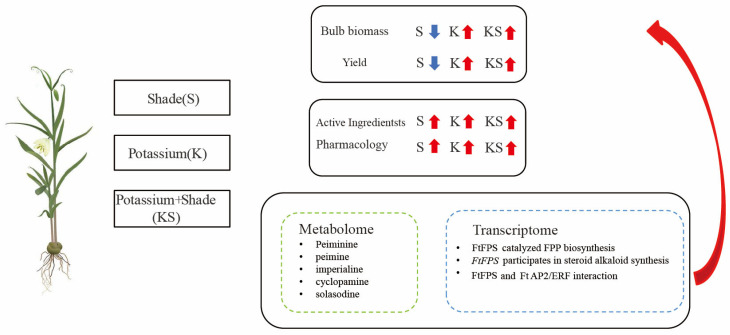
Model of the influence of potassium application and shading conditions on the biosynthesis of steroidal alkaloids in *F. thunbergii*. Red represents increase and blue represents decrease.

Although shading reduces the *F. thunbergii* bulb biomass and yield, it markedly increases its active ingredient content and accumulation. The combined application of shading and potassium fertilizer not only greatly alleviates the biomass and yield losses caused by shading but also significantly improves the active ingredient content and accumulation in the bulbs of *F. thunbergii*. The aim of these comprehensive metabolomics and transcriptomics analyses was to determine the intricate mechanisms by which potassium application and shading conditions influence steroidal alkaloid biosynthesis in *F. thunbergii* bulbs. Peiminine, peimine, imperialine, solasodine, and cyclopamine represent the predominant classes of steroidal alkaloids found in the *F. thunbergii* bulb. In two pivotal signaling pathways (MVA and MEP), *FTFPS* emerges as the essential structural gene. Moreover, the FtAP2/ERF TF family exhibits a positive correlation with steroidal alkaloid biosynthesis. Moreover, the *FtFPS* gene is the key gene in the co-expression network, and the Y2H experiment showed that it interacts with FtAP2/ERF. The *FtFPS* gene facilitates the production of FPP. This study sheds additional light on the synthesis of steroidal alkaloids in the *F. thunbergii* bulb by integrating transcriptome and metabolome analyses (Figure 9).

## Data Availability

The unprocessed information acquired from the sequencing procedure has been kept in the Sequence Read Archive (SRA) at the National Center for Biotechnology Information(NCBI) repository, identifiable by the accession number SRR31178335 through SRR31178358.

## Supplementary Materials

The following supporting information can be downloaded at: https://www.mdpi.com/article/10.3390/biology14060633/s1, Figure S1. Effects of shading on photosynthetically active radiation (PAR) of *F. thunbergii* plants in 2023. Values are the means ± SE, n = 6; Figure S2. The base peak chromatogram of the samples in positive ion mode (A) and negative ion mode (B); Figure S3. Principal component analysis (PCA) of the metabolite qualification in *F. thunbergii* bulb in response to different conditions. (A) PCA results derived from LC-MS/MS (ESI+). (B) PCA results derived from LC-MS/MS (ESI-); Figure S4. Correlation analysis between metabolites in 24 samples. High and low levels are depicted using red and blue scales, respectively; Figure S5. Composition of metabolites in *F. Thunbergii* bulbs from different treatments based on superclass; Figure S6. PLS-DA of metabolites in *F. Thunbergii* bulbs from different treatments for (A) positive ion mode and (B) negative ion mode, respectively; Figure S7. The PLS-DA permutation test for the (A) positive ion mode and (B) negative ion mode, respectively; Figure S8. DAMs in *F. thunbergii* bulb in response to different conditions. (A) Numbers of DAMs of *F. thunbergii* bulb under different treatments. (B) Venn diagram of DAMs in *F. thunbergii* bulb; Figure S9. Structural confirmation was conducted by comparison with the reference standards or matching with theoretical data or commercial library; Figure S10. Principal component analysis of *F. thunbergii* bulb transcriptomes in response to different conditions; Figure S11. SDS-PAGE analysis of the expression of FtFPS. M: Marker, pET32a: intracellular soluble of pET32a; pET32a-FtFPS: intracellular soluble of pET32a-FtFPS; Table S1. Name and sequence of primer pairs; Table S2. All annotated metabolites in *F. thunbergii* bulb in response to different conditions; Table S3. Summary of RNA-Seq reads after filtering; Table S4. Different expressed genes related to *F. thunbergii* in response to different conditions.

## Abbreviations

The following abbreviations are used in this manuscript:

HMGR	3-hydroxy-3-methylglutaryl coenzyme A reductase
MK	mevalonate kinase
FPS	farnesyl pyrophosphate synthase
DXR	1-deoxy-D-xylulose-5-phosphate reductoisomerase
MCS	2-C-methyl-D-erythritol 2,4-cyclodiphosphate synthase
AOP2	antioxidant protein 2
DMAPP	dimethylallyl diphosphate
IPP	isopentenyl diphosphate
MVD	mevalonate diphosphate decarboxylase
AACT	acetyl-CoA acetyltransferase
DOXP	1-deoxy-D-xylulose-5-phosphate
G3P	glyceraldehyde-3-phosphate
HMGS	3-hydroxy-3-methylglutaryl-CoA synthase
PMK	phosphomevalonate kinase
HDS	1-hydroxy-2-methyl-2-butenyl-4-diphosphate synthase
MCT	2-C-methyl-D-erythritol-4-phosphate cytidylyltransferase
CMK	4-diphosphocytidyl-2-C-methyl-D-erythritol kinase
FPP	farnesyl pyrophosphate
SQS	squalene synthase
SQE	squalene epoxidase
SSR2	sterol side chain reductase2
3βHSD	3-beta-hydroxysteroid-dehydrogenase
CPI	cyclopropyl sterol isomerase
CYP734A6	cytochrome P450 734A6
CYP94N2	cytochrome P450 94N2
GAME17	gamma-solanine acetyltransferase 17
MVA	Mevalonic acid
MEP	Methylerythritol phosphate
AHS	Nin Jiom Pei Pa Koa
KNHRCPS	Ambroxol Hydrochloride
DSP	Dexamethasone Sodium Phosphate Injection
PBS	Phosphate-Buffered Saline
GC-MS	Gas Chromatography–Mass Spectrometry
PLS-DA	Partial Least Squares-Discriminant Analysis
