# Supplementary material for: Regulation of Steroidal Alkaloid Biosynthesis in Bulbs of Fritillaria thunbergii Miq. By Shading and Potassium Application: Integrating Transcriptomics and Metabolomics Analyses
Source: Biology (Basel). 2025 May 29;14(6):633. doi: 10.3390/biology14060633 (PMC12189873; doi:10.3390/biology14060633)
Supplement: Supplementary file 1 [file biology-14-00633-s001.zip › Supplementary figures.pdf]

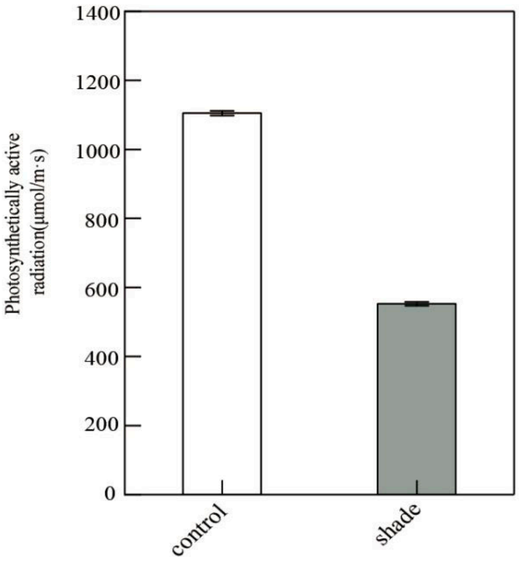

Fig S1. Effects of shading on photosynthetically active radiation (PAR) of *F. thunbergii* plants in 2023. Values are the means ± SE, n=6.

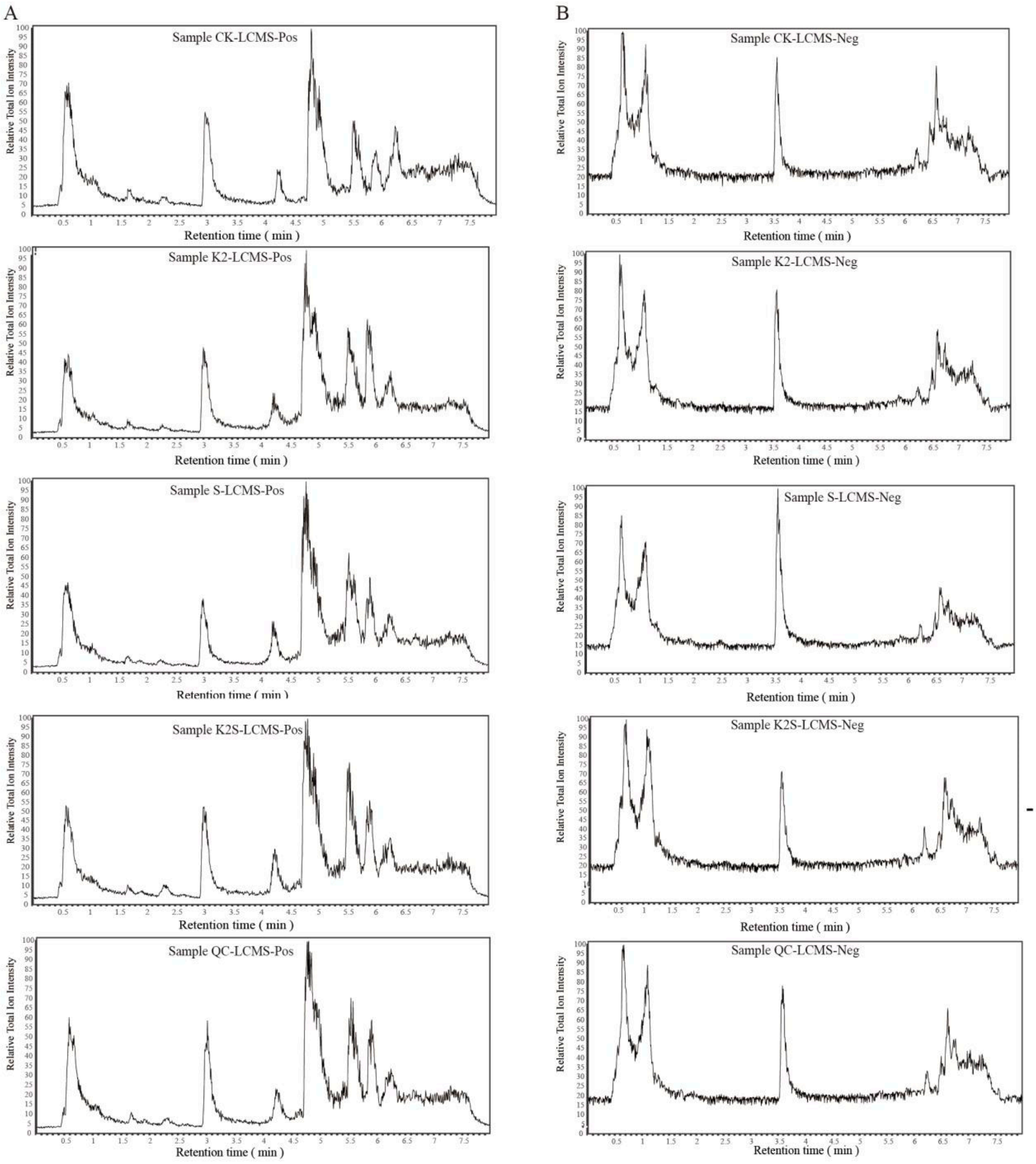

Figure S2. The base peak chromatogram of the samples in positive ion mode (A) and negative ion mode (B).

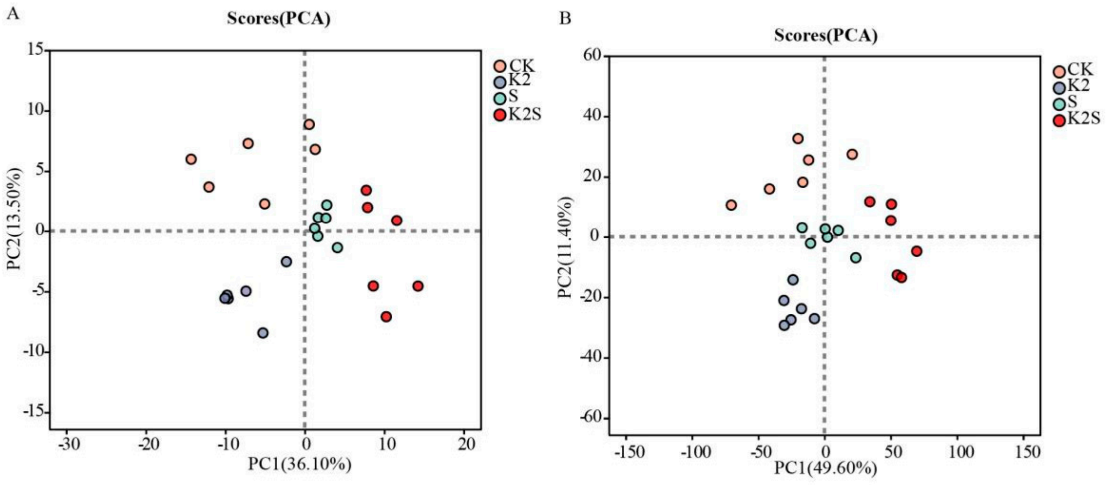

Fig S3. Principal component analysis (PCA) of the metabolite qualification in *F. thunbergii* bulb in response to different conditions. (A) PCA results derived from LC-MS/MS (ESI+). (B) PCA results derived from LC-MS/MS (ESI-).

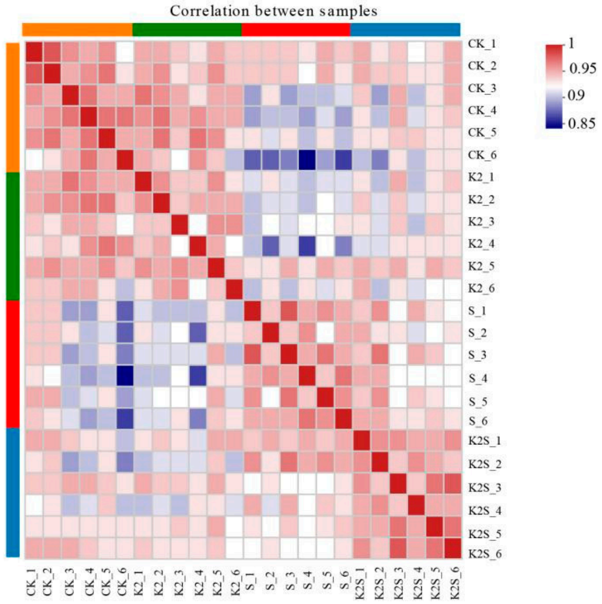

Fig S4. Correlation analysis between metabolites in 24 samples. High and low levels are depicted using red and blue scales, respectively.

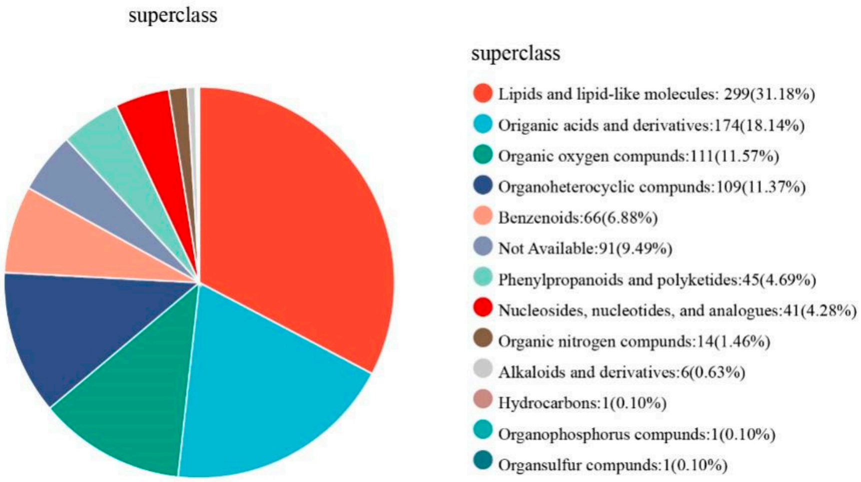

Figure S5. Composition of metabolites in *F. Thunbergii* bulbs from different treatments based on superclass.

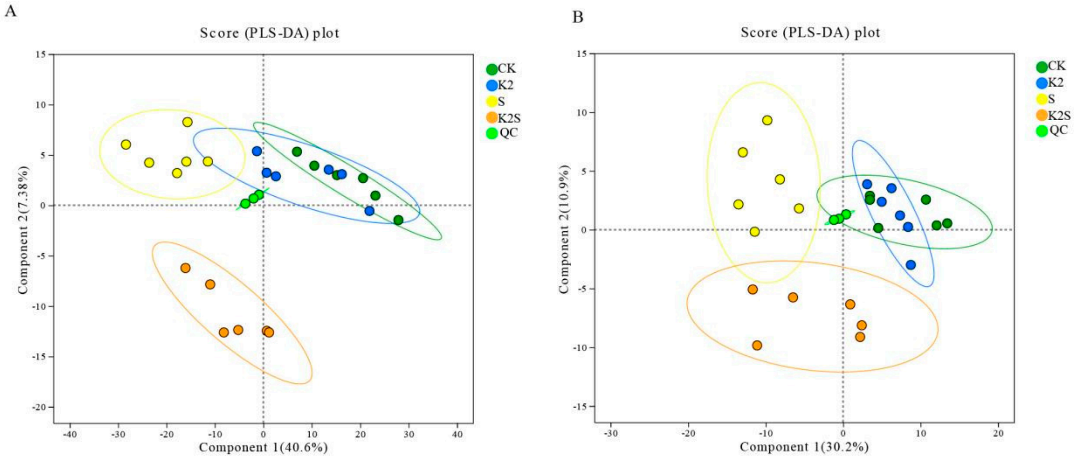

Figure S6. PLS-DA of metabolites in *F. Thunbergii* bulbs from different treatments for (A) positive ion mode and (B) negaive ion mode, respectively.

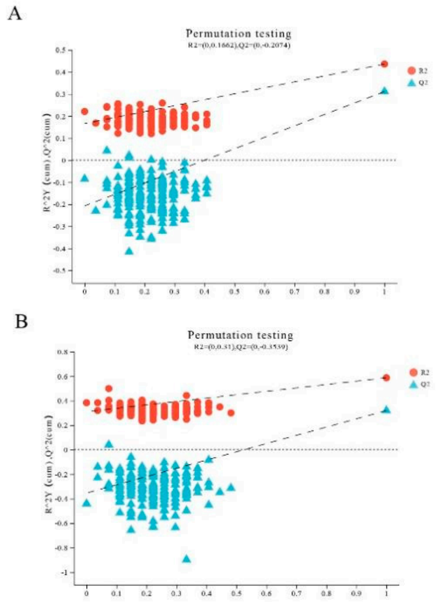

Figure S7. The PLS-DA permutation test for the (A) positive ion mode and (B) negative ion mode, respectively.

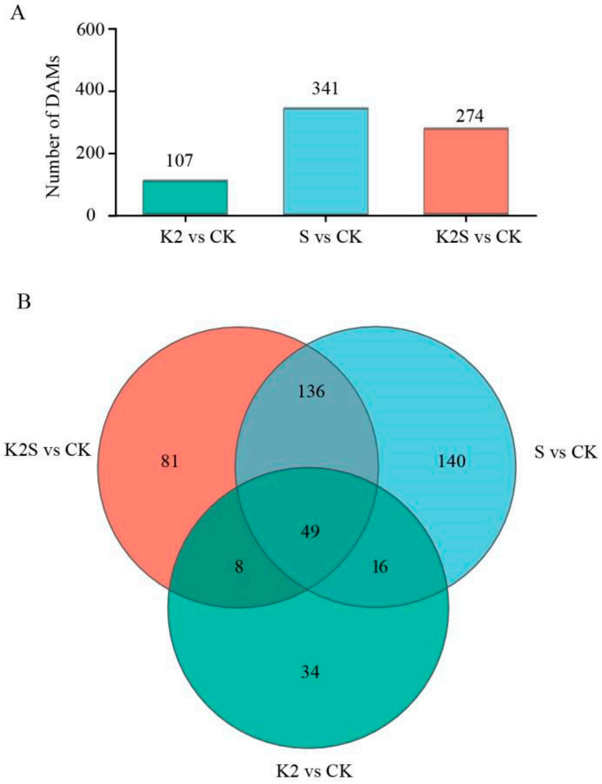

Fig S8. DAMs in *F. thunbergii* bulb in response to different conditions. (A) Numbers of DAMs of *F. thunbergii* bulb under different treatments. (B) Venn diagram of DAMs in *F. thunbergii* bulb.

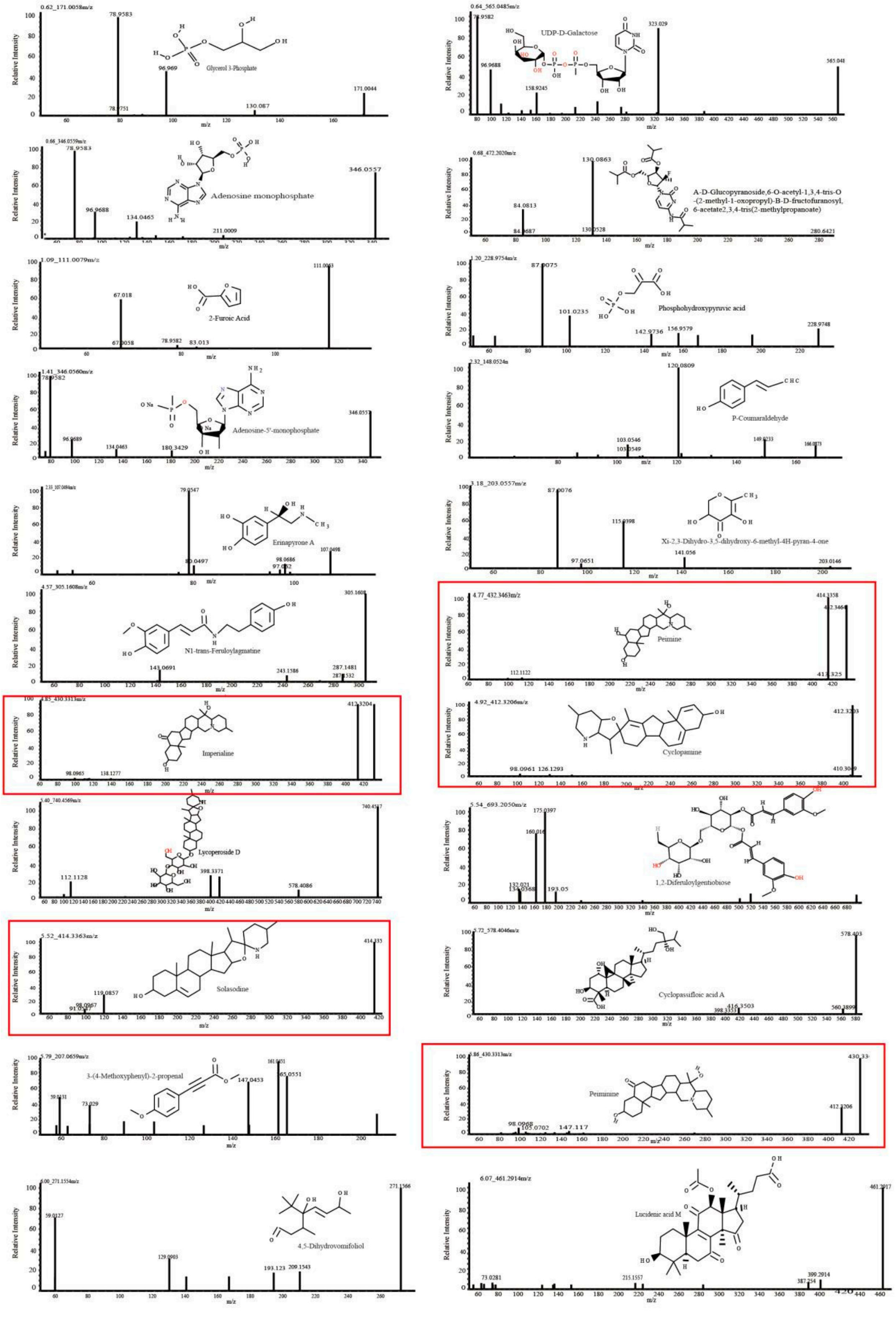

Figure S9. Structural confirmation was conducted by comparison with the reference standards or matching with theoretical data or commercial library.

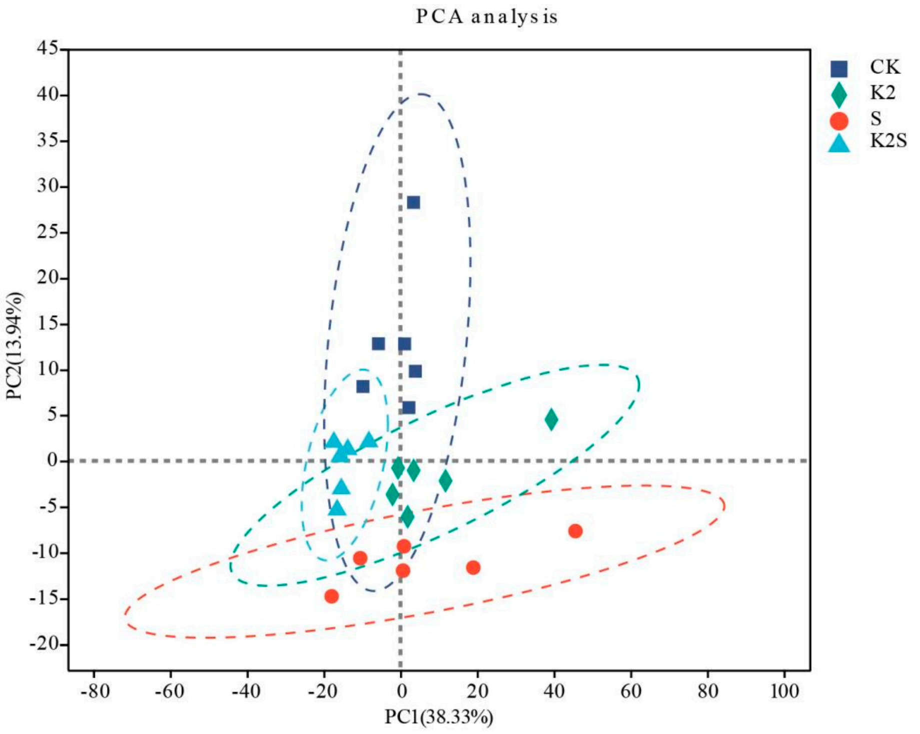

Fig S10. Principal component analysis of *F. thunbergii* bulb transcriptomes in response to different conditions.

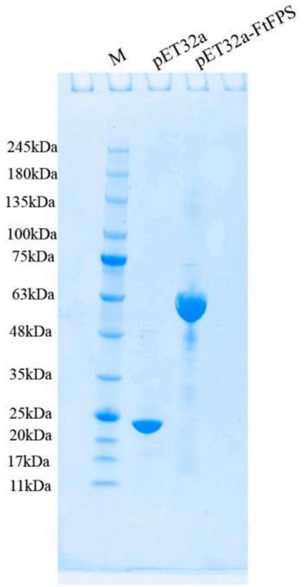

Fig S11. SDS-PAGE analysis of the expression of FtFPS. M: Marker, pET32a: intracellular soluble of pET32a; pET32a-FtFPS: intracellular soluble of pET32a-FtFPS.
