# Supplementary figures and images for: Regulation of Steroidal Alkaloid Biosynthesis in Bulbs of Fritillaria thunbergii Miq. By Shading and Potassium Application: Integrating Transcriptomics and Metabolomics Analyses
Source: Biology (Basel). 2025 May 29;14(6):633. doi: 10.3390/biology14060633 (PMC12189873; doi:10.3390/biology14060633)

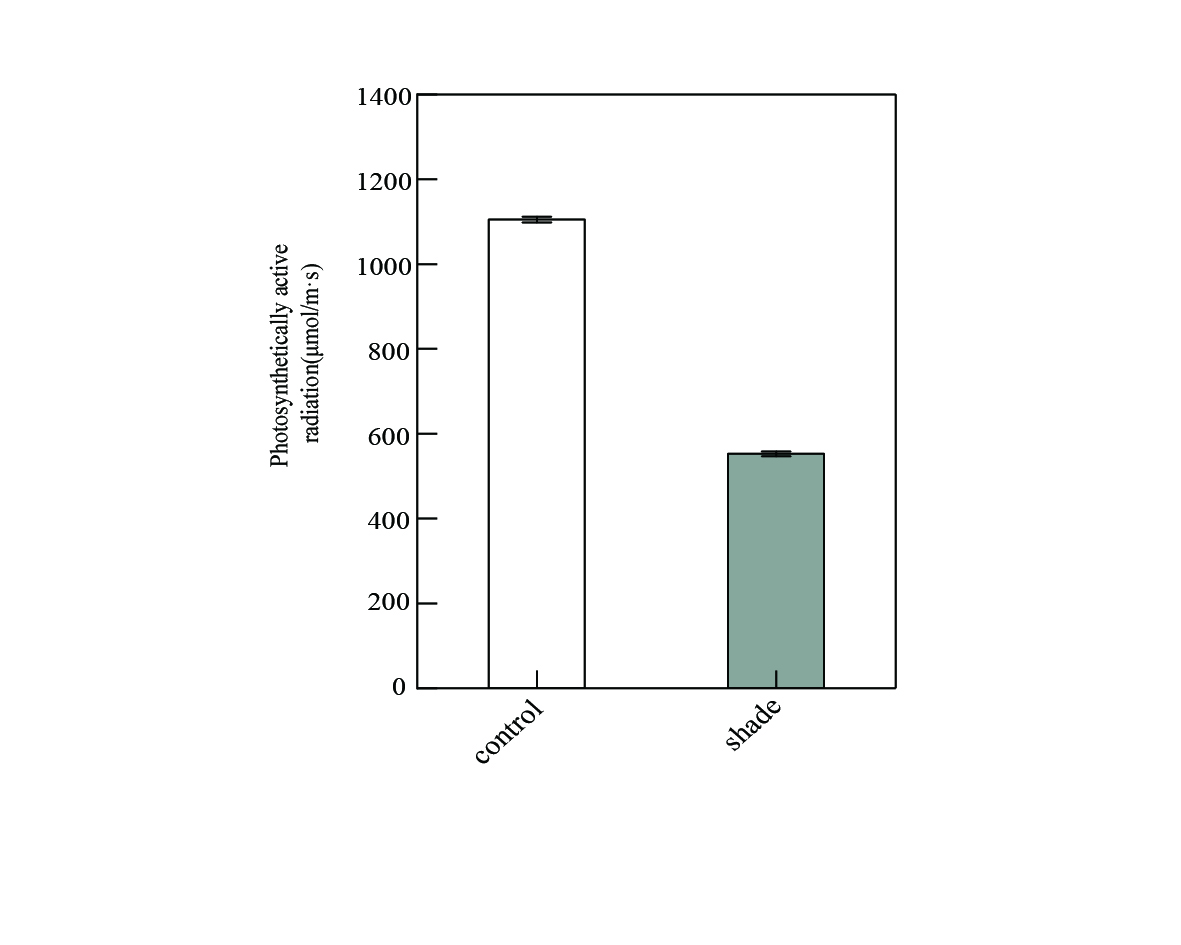

Supplement: Supplementary file 1 [file biology-14-00633-s001.zip › Figure S1.jpg]

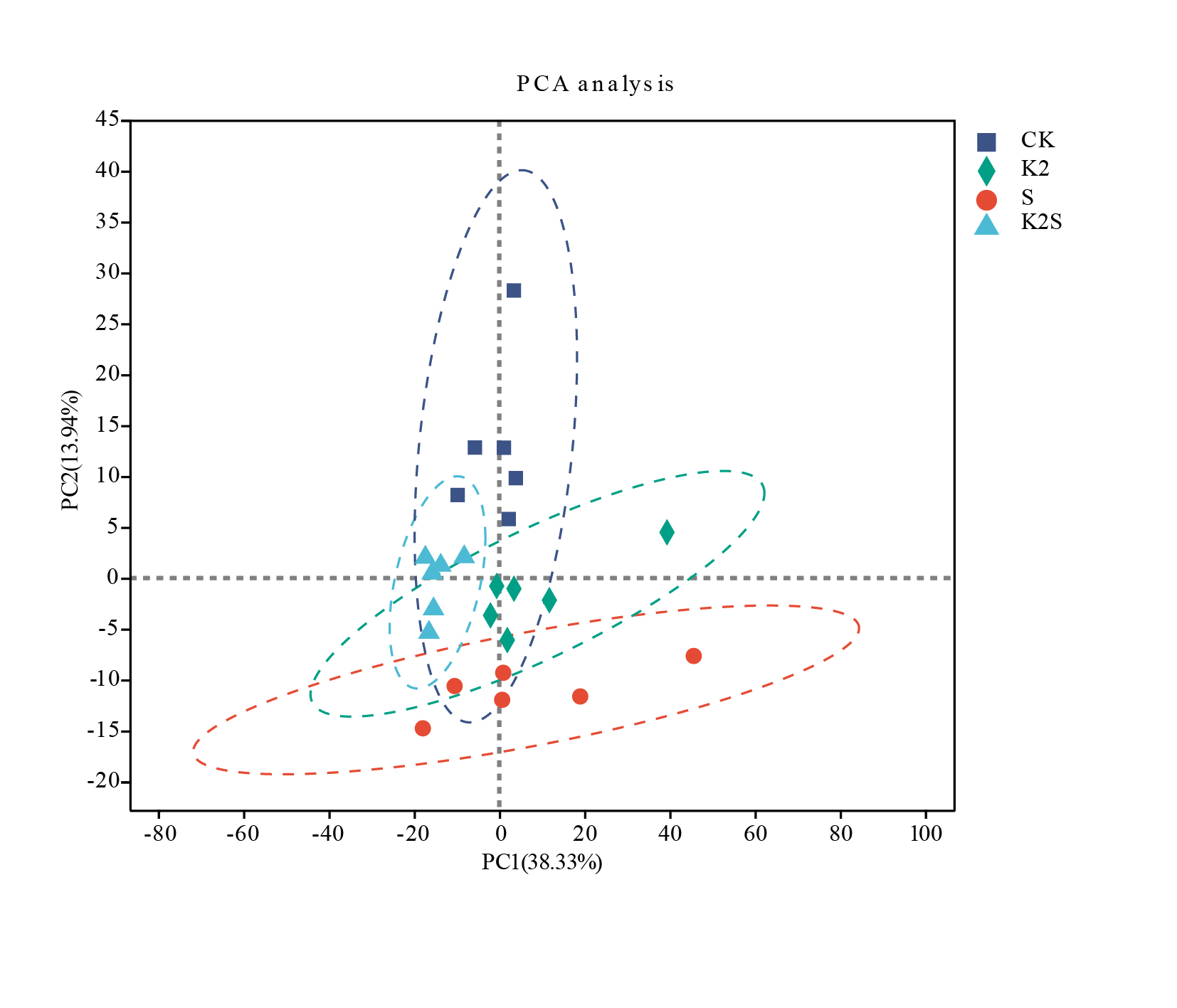

Supplement: Supplementary file 1 [file biology-14-00633-s001.zip › Figure S10.jpg]

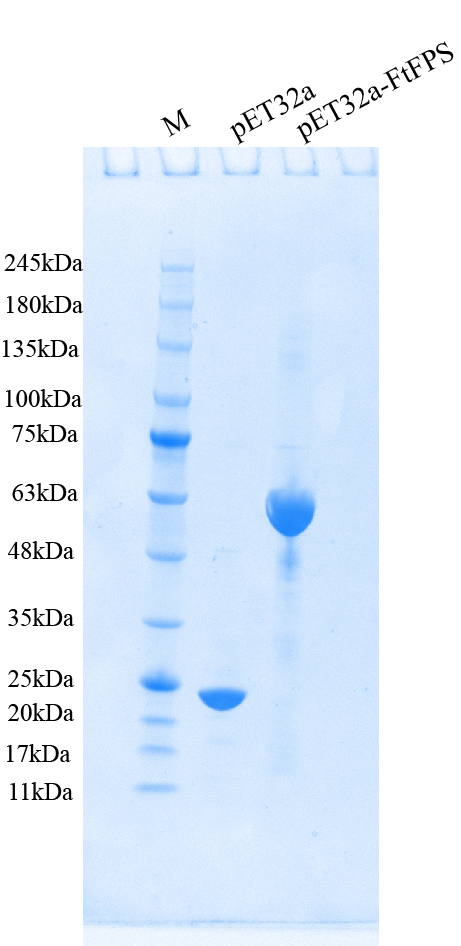

Supplement: Supplementary file 1 [file biology-14-00633-s001.zip › Figure S11.jpg]

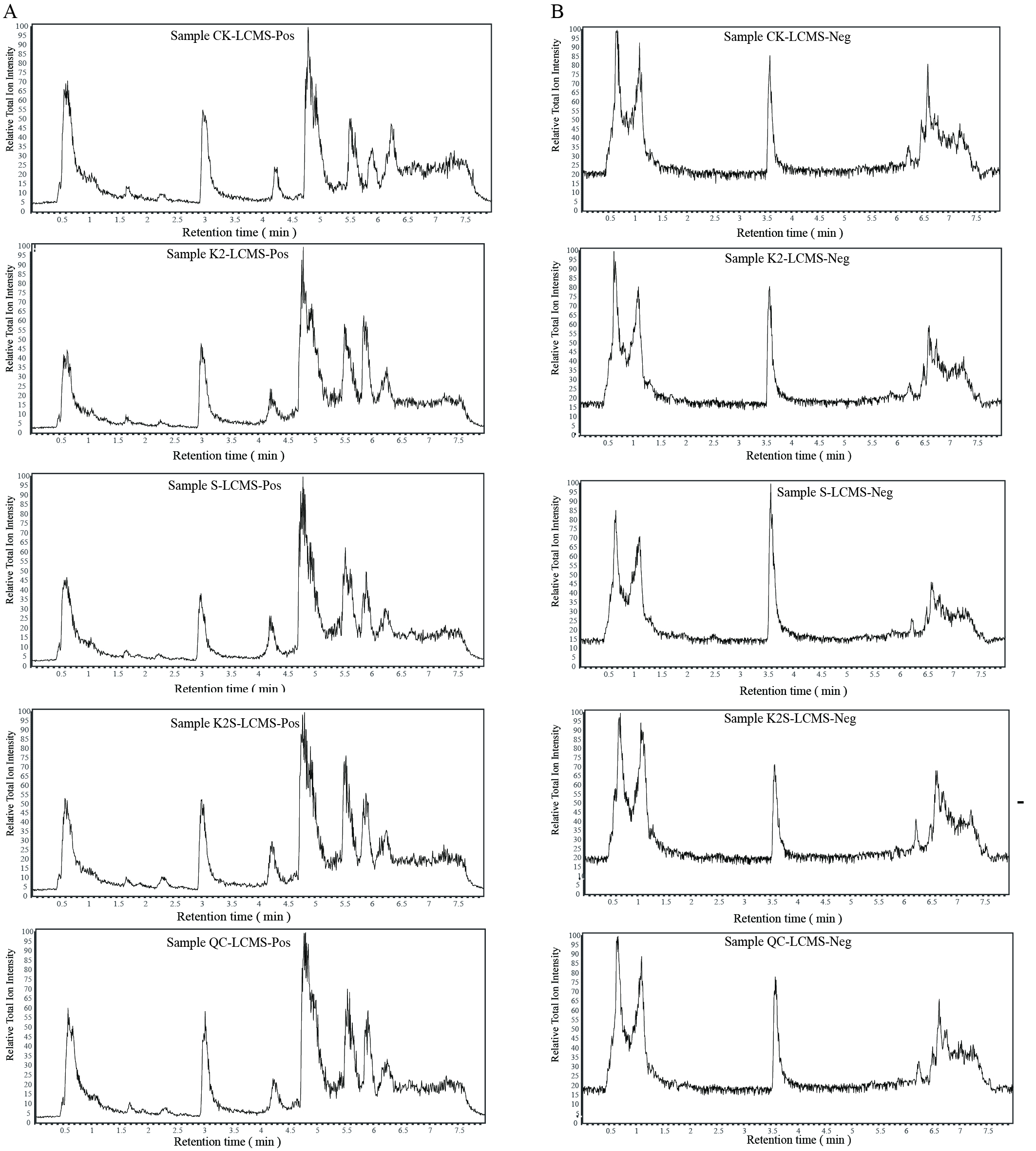

Supplement: Supplementary file 1 [file biology-14-00633-s001.zip › Figure S2.jpg]

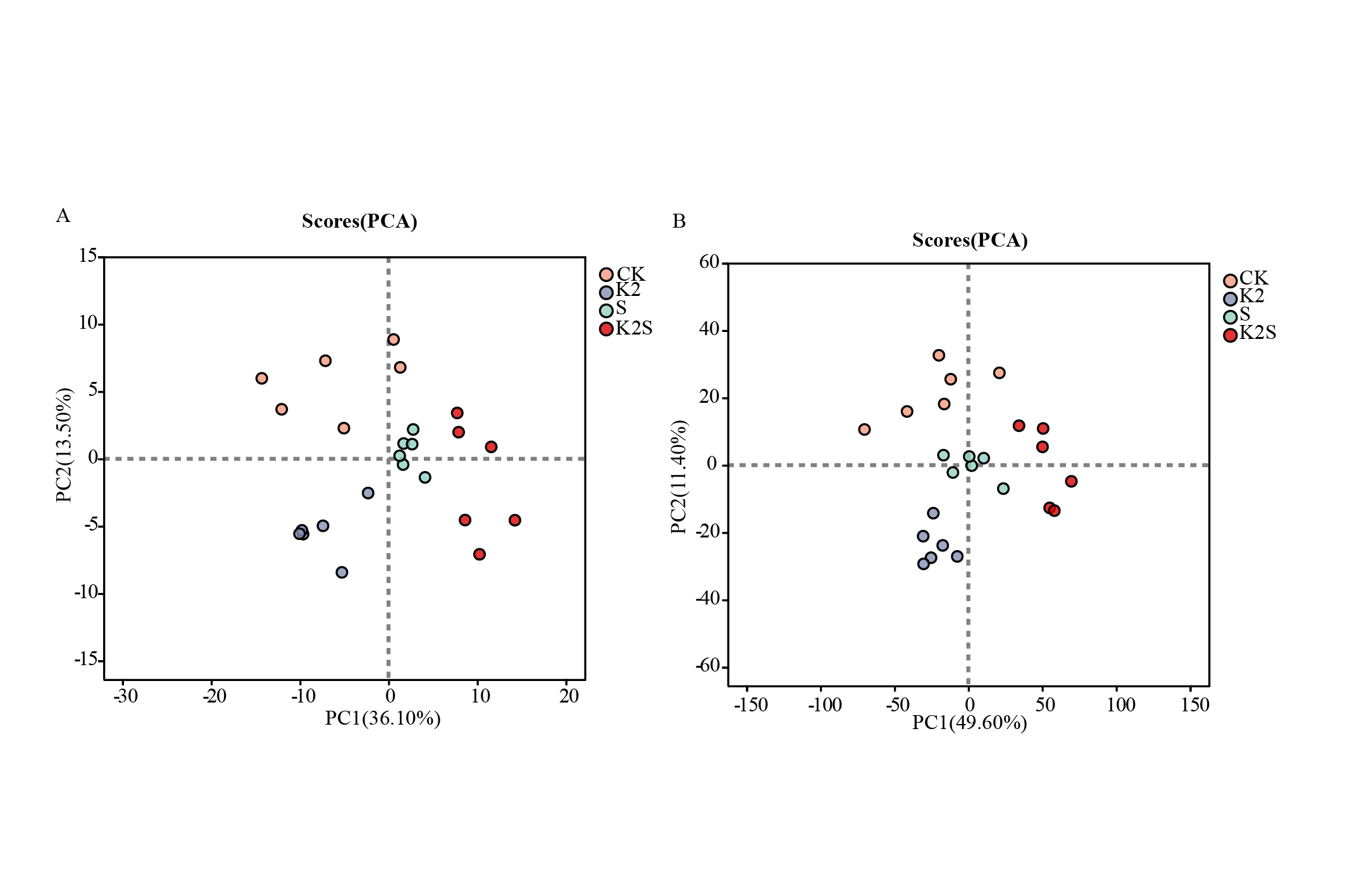

Supplement: Supplementary file 1 [file biology-14-00633-s001.zip › Figure S3.jpg]

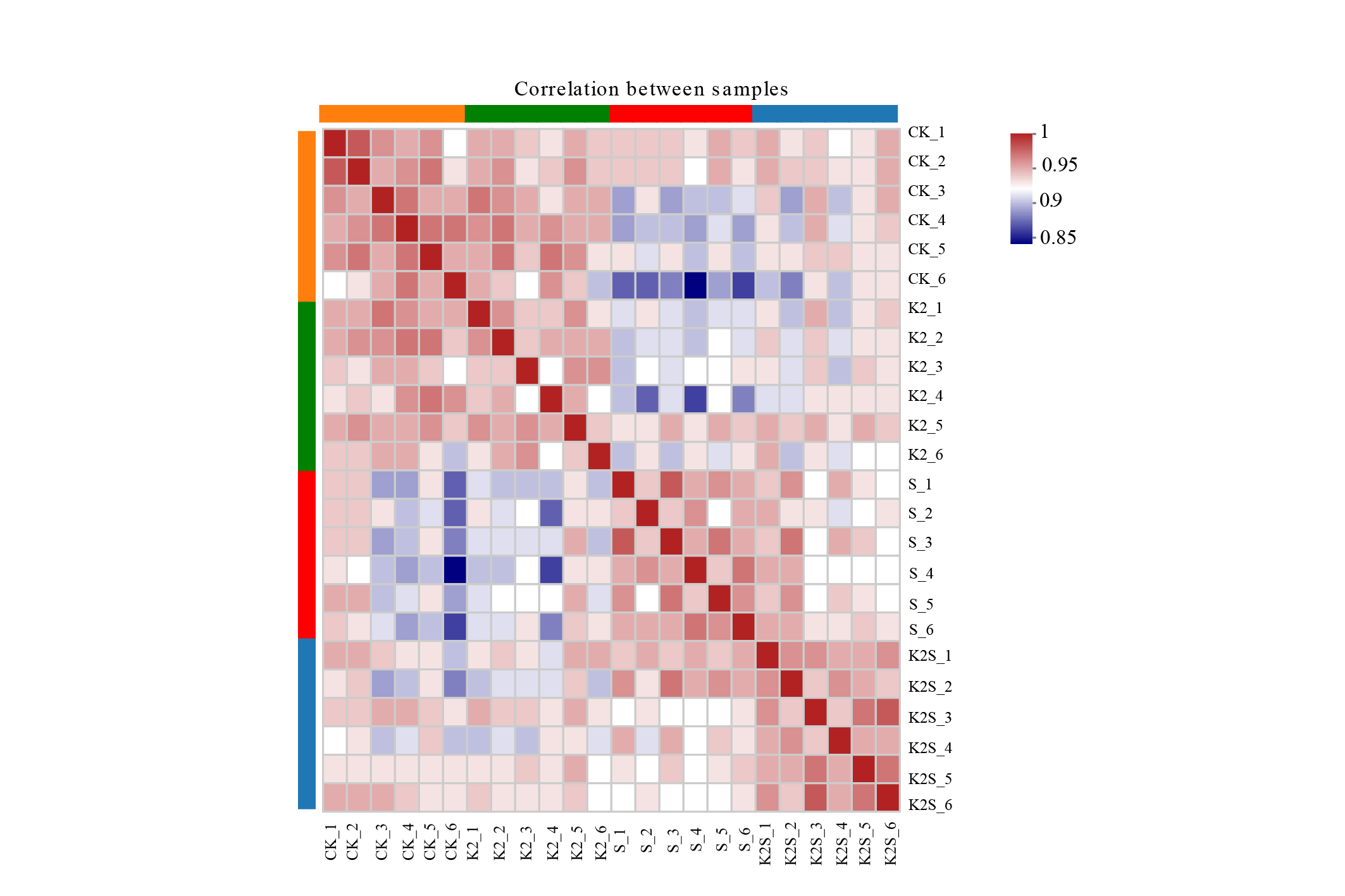

Supplement: Supplementary file 1 [file biology-14-00633-s001.zip › Figure S4.jpg]

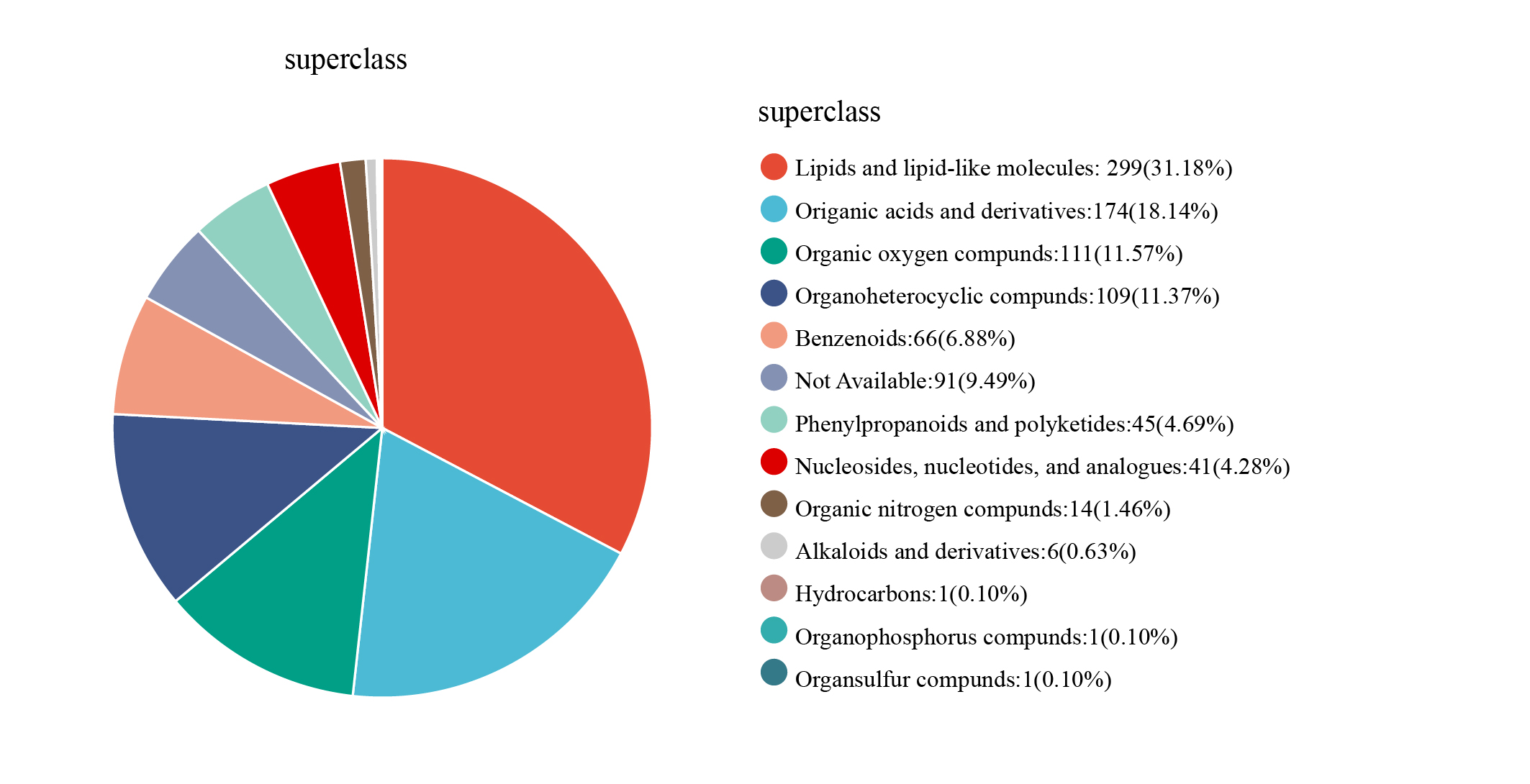

Supplement: Supplementary file 1 [file biology-14-00633-s001.zip › Figure S5.jpg]

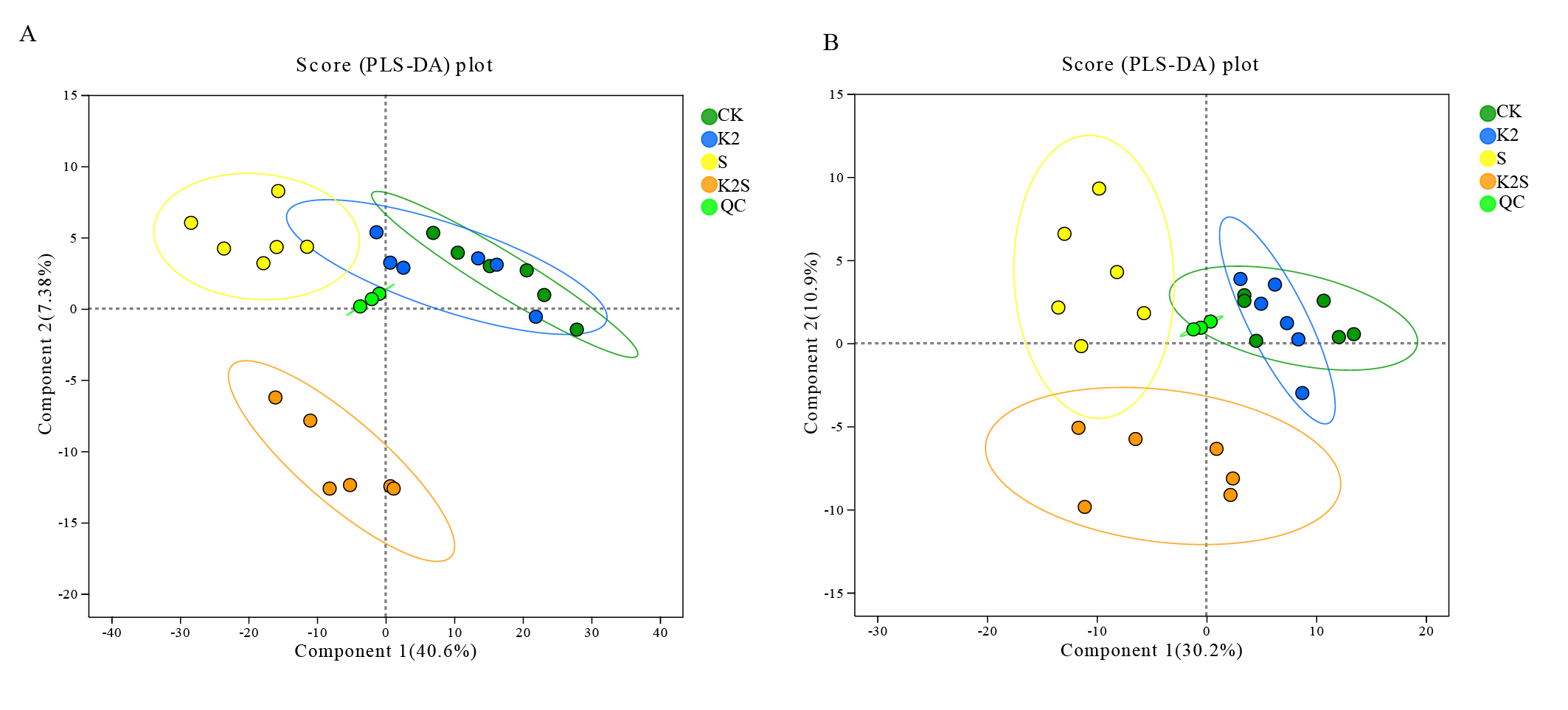

Supplement: Supplementary file 1 [file biology-14-00633-s001.zip › Figure S6.jpg]

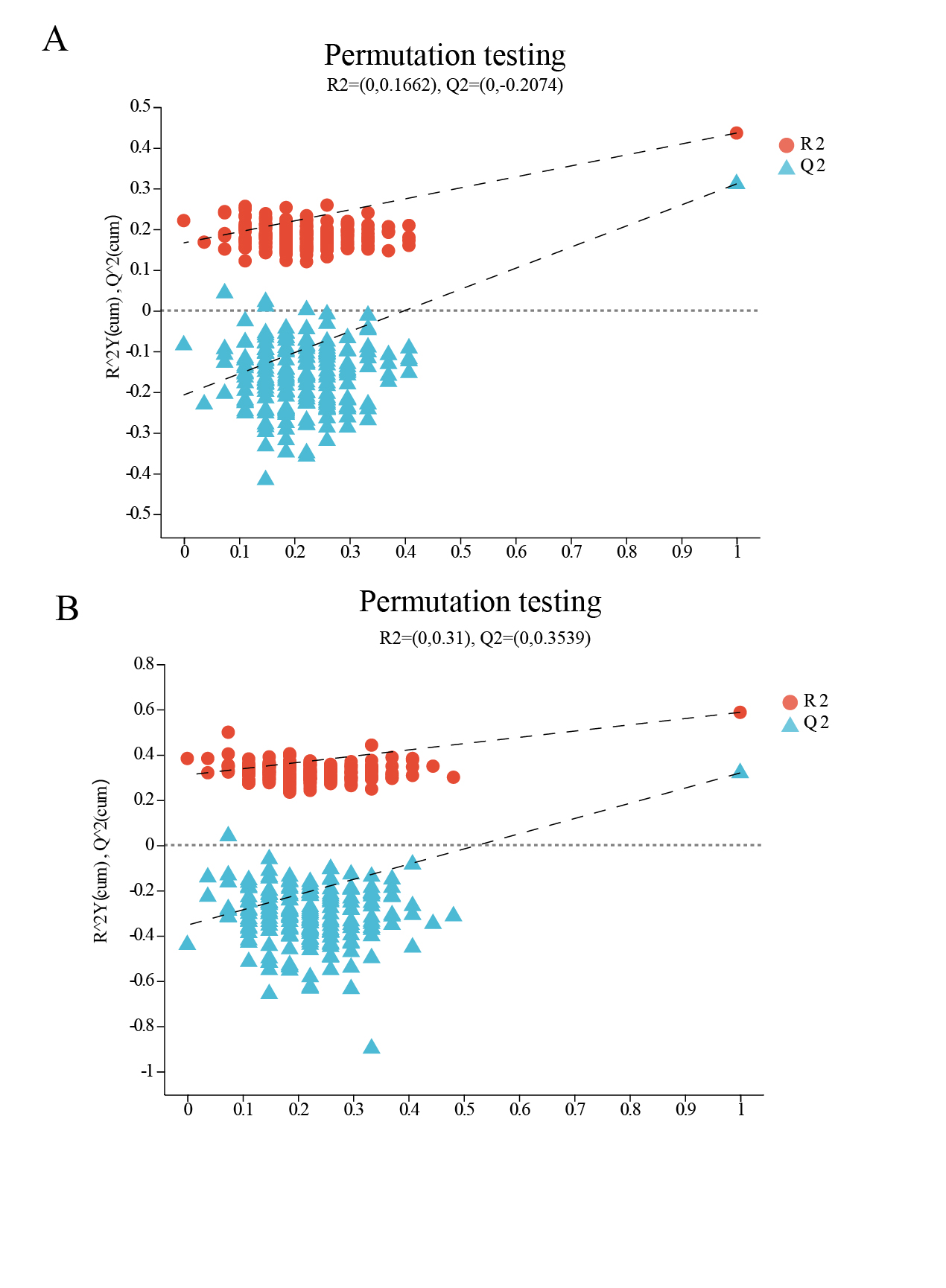

Supplement: Supplementary file 1 [file biology-14-00633-s001.zip › Figure S7.jpg]

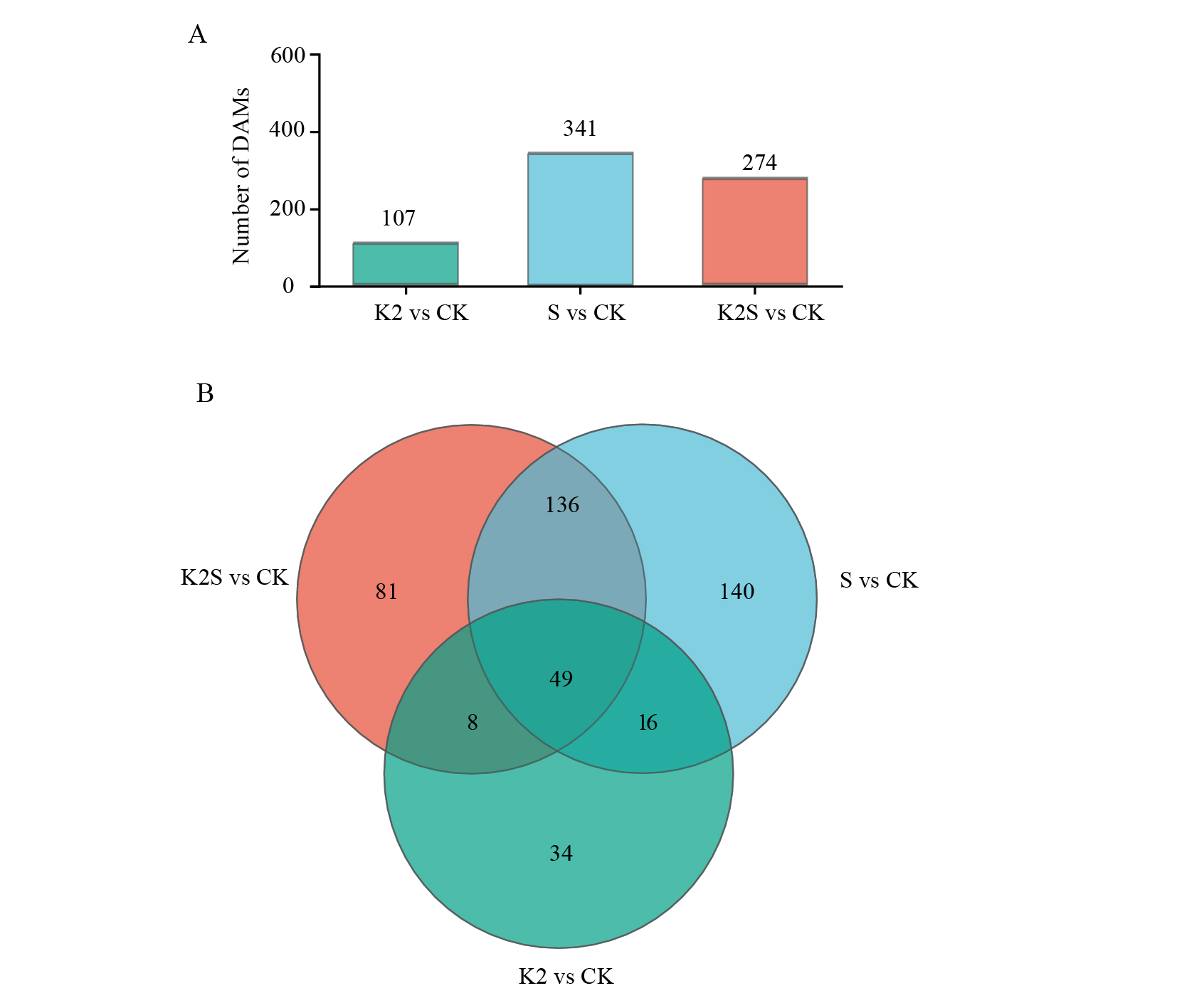

Supplement: Supplementary file 1 [file biology-14-00633-s001.zip › Figure S8.jpg]

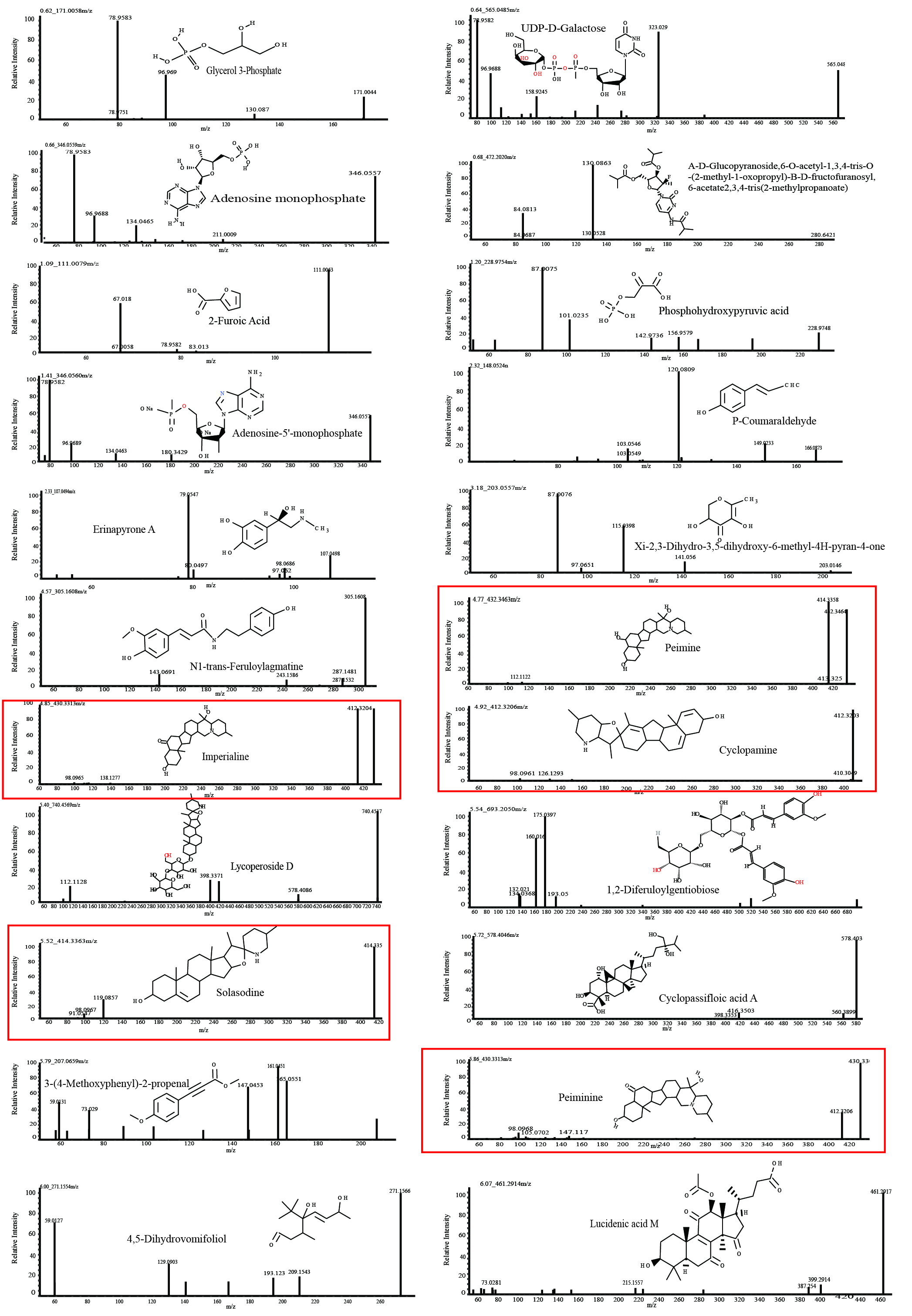

Supplement: Supplementary file 1 [file biology-14-00633-s001.zip › Figure S9.jpg]
